# Quantitative Expression Profile of Distinct Functional Regions in the Adult Mouse Brain

**DOI:** 10.1371/journal.pone.0023228

**Published:** 2011-08-12

**Authors:** Takeya Kasukawa, Koh-hei Masumoto, Itoshi Nikaido, Mamoru Nagano, Kenichiro D. Uno, Kaori Tsujino, Carina Hanashima, Yasufumi Shigeyoshi, Hiroki R. Ueda

**Affiliations:** 1 Functional Genomics Unit, RIKEN Center for Developmental Biology, Kobe, Hyogo, Japan; 2 Laboratory for Systems Biology, RIKEN Center for Developmental Biology, Kobe, Hyogo, Japan; 3 Department of Anatomy and Neurobiology, Kinki University School of Medicine, Osaka, Japan; 4 Graduate School of Science, Osaka University, Osaka, Japan; 5 Laboratory for Neocortical Development, RIKEN Center for Developmental Biology, Hyogo, Japan; 6 Department of Mathematics, Graduate School of Science, Kyoto University, Kyoto, Japan; 7 Laboratory for Synthetic Biology, RIKEN Quantitative Biology Center, Kobe, Hyogo, Japan; National Institutes of Health, United States of America

## Abstract

The adult mammalian brain is composed of distinct regions with specialized roles including regulation of circadian clocks, feeding, sleep/awake, and seasonal rhythms. To find quantitative differences of expression among such various brain regions, we conducted the BrainStars (B*) project, in which we profiled the genome-wide expression of ∼50 small brain regions, including sensory centers, and centers for motion, time, memory, fear, and feeding. To avoid confounds from temporal differences in gene expression, we sampled each region every 4 hours for 24 hours, and pooled the samples for DNA-microarray assays. Therefore, we focused on spatial differences in gene expression. We used informatics to identify candidate genes with expression changes showing high or low expression in specific regions. We also identified candidate genes with stable expression across brain regions that can be used as new internal control genes, and ligand-receptor interactions of neurohormones and neurotransmitters. Through these analyses, we found 8,159 multi-state genes, 2,212 regional marker gene candidates for 44 small brain regions, 915 internal control gene candidates, and 23,864 inferred ligand-receptor interactions. We also found that these sets include well-known genes as well as novel candidate genes that might be related to specific functions in brain regions. We used our findings to develop an integrated database (http://brainstars.org/) for exploring genome-wide expression in the adult mouse brain, and have made this database openly accessible. These new resources will help accelerate the functional analysis of the mammalian brain and the elucidation of its regulatory network systems.

## Introduction

The adult mammalian brain is one of the most sophisticated and complex organs devised by nature. The distinct functional regions that comprise it are responsible for processing internal and external information into the panoply of mammalian experience. The different locations in the adult brain have specialized functions, and various kinds of brain “maps” (or atlases), including anatomical and functional maps [1], [2], [3], [4], have been developed to illustrate them. Recently, “expression” brain maps, showing the gene transcription profiles of different brain regions, have been constructed. Since the distinct anatomical structures of the brain and their functions develop from and are regulated by transcription, at least in part, expression maps should, to some extent, delineate the same brain regions. In fact, this idea is supported by the results of published brain transcription profiles [5], [6], [7], [8], [9], [10], [11], [12], [13], [14], [15], [16], [17], which show concordance between gene transcription and anatomical and functional brain regions. To obtain expression maps of various brain regions, *in situ* hybridization (ISH) methods have been widely used, and recently, genome-wide collections of ISH data have been created [5], [6], [7], [18], including the EMAGE (Edinburgh Mouse Atlas Gene Expression Database) [19], GenePaint [6], BGEM (St. Jude Brain Gene Expression Map) [8], BrainMaps.org [20], and Allen Brain Atlas [9]. Although the expression data obtained by ISH can provide good, cellular-level resolution in sliced surfaces, its signals have a narrow dynamic range [21], which can hinder relative comparisons of expression levels between brain regions. DNA-microarray technology is an alternative way to obtain quantitative genome-wide expression data in tissues and cell culture [22], [23]. This technology is widely used in biological research, including in neuroscience, and several groups have published resources showing transcript expression profiles in areas of the mammalian brain [10], [11], [12], [13], [14], [15], [16], [17]. Although these resources provide quantitative expression data, the size of each sampled region is relatively large to ensure that adequate volumes of RNA samples are collected, and therefore, multiple functional nuclei, loci, ganglia, or substantia are merged into a single sampled region. Therefore, no single approach can satisfy quantitativeness and spatial resolution simultaneously even at the nucleus-level.

To achieve a good balance between quantitativeness and spatial resolution for an expression profile of distinct functional regions in the adult mouse brain, we attempted a two-step approach. We first obtained the expression data of nucleus-level resolution in the adult mouse brain as a primary data resource, by using DNA-microarray technology. Although nucleus-level resolution adopted in this study is larger than cellular-level resolution, the nucleus-level expression profile can still provide useful information to identify the genes whose expression are changed in a target brain region related to a specific function (e.g. food intake or circadian and photoperiodic behavior), or to identify the brain regions where a gene of interest has differential expression. The information of identified genes or brain regions can be used to plan, for example, construction of knock-out or knock-in mouse or further inspection of cellular-level ISH datasets. Therefore, as a second step, we integrated the primary expression data obtained with the various existing mouse brain expression maps including ISH expression data of cellular-level resolution. We call this entire project (nucleus-level, quantitative expression profiling as well as construction of integrated web interface) as the BrainStars (B*) project. We then used informatics to analyze the spatial and quantitative genome-wide expression patterns in distinct functional brain regions. The entire BrainStars dataset is publicly available through the integrated database (http://brainstars.org/).

## Results

### Quantitative expression profile of the brain

**Figure 1:**
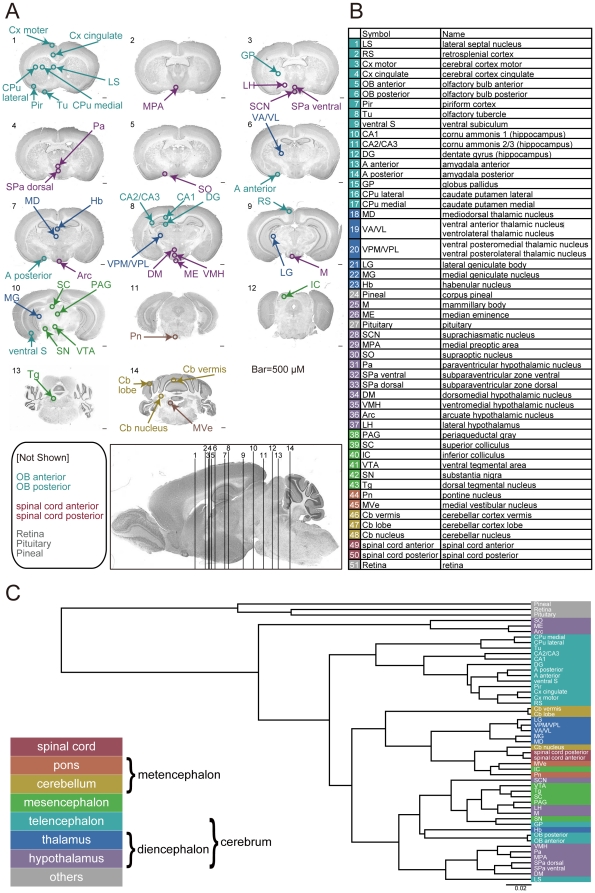
Sampled adult mouse CNS regions. (**A**) Map of 44 of the 51 sampled CNS regions. The other seven regions are listed. (**B**) Abbreviations and full names of CNS regions. (**C**) Hierarchical clusters of the expression profiles of the 51 CNS regions. Background colors indicate the classical developmental/evolutional/anatomical classification of each region.

We sampled 51 regions with distinct functions in the central nervous system (CNS) of the adult mouse: 49 brain regions and 2 spinal-cord regions (**Figure 1A–B** and **Table S1**), including centers of vision, hearing, taste, olfaction, touch, motion, clocks, calendar, memory, fear, and feeding. We used cylindrical punch samples, 0.5-mm thick and 0.5 mm in diameter, from 51 distinct CNS regions. To ensure the accuracy of our spatial expression dataset, we took samples of the CNS regions every 4 hours, starting at ZT0 (Zeitgaber time 0; the time of lights on), for 24 hours (6 time-point samples for each region) to avoid potential artifacts caused by the circadian regulation of gene expression. We sampled 5–25 mice for each CNS region at each time point (every 4 hours during one day), resulting in samples from 30–150 mice being collected for each replicate of a single CNS region. This entire procedure was performed twice (n = 2) to obtain experimental replicates for every CNS region. The sample quality was confirmed before carrying out the DNA-microarray experiments by the quantitative PCR (q-PCR) analysis of known region-specific genes. After the DNA-microarray experiments, we also confirmed the sample quality by the visual inspection of quality-check graphs, such as global sample clustering (**Figure S1A–B**) and degradation plots [24]. This sampling strategy and quality check of samples helped to reduce the expression variability between experimental replicates of the same brain region, as evidenced by the high correlation (0.994) between experimental replicates (**Figure S1F–G**). We also noted that most experimental replicates were clustered together (**Figure S1A–B**). Even for mis-clustered replicates of CNS regions (e.g. the cerebral cortex motor and cerebral cortex cingulate), when we used an appropriate set of regional marker genes (e.g. *Myl4*) retrieved from other *in situ* databases, their expression patterns in our data resource could correctly distinguish them. These results support the reproducibility and consistency of the data in the BrainStars project.

### Global clustering of CNS regions

For the first expression data analysis, we performed global clustering of these 51 CNS regions for all the DNA microarray probe sets (45,037) except for the controls, using different distance metrics (**Figure 1C** and **Figure S1C**). The clustering results indicated that most of the 51 regions were grouped according to their developmental/evolutional/anatomical classifications independently of the distance metrics; for example, into the telencephalon, thalamus, hypothalamus, or mesencephalon. Interestingly, some regions did not cluster according to their classical developmental/evolutional/anatomical classifications, and these exceptional clusters seemed to represent more recent evolutionary-developmental processes supporting their sophisticated functional linkage. For example, the globus pallidus (GP) and substantia nigra (SN), which are classified into different classical developmental/evolutional/anatomical divisions, the telencephalon and mesencephalon, respectively, but are functionally and anatomically linked, exhibit a tight and robust clustering of their genome-wide expressions in correlation and Euclidean distance (**Figure 1C** and **Figure S1C–E;** see also **Text S1**). We also noted that three CNS regions (the retina, pituitary, and pineal) were significantly separated from the other 48 in the global clustering analysis (**Figure 1C** and **Figure S1C–E**), possibly owing to their anatomical differences or different proportions of multiple cell types. Therefore, we used only the other 48 regions for our further analysis in the following sections, to focus on subtler differences in gene expression.

### “Multi-state” expression patterns in CNS regions

**Figure 2:**
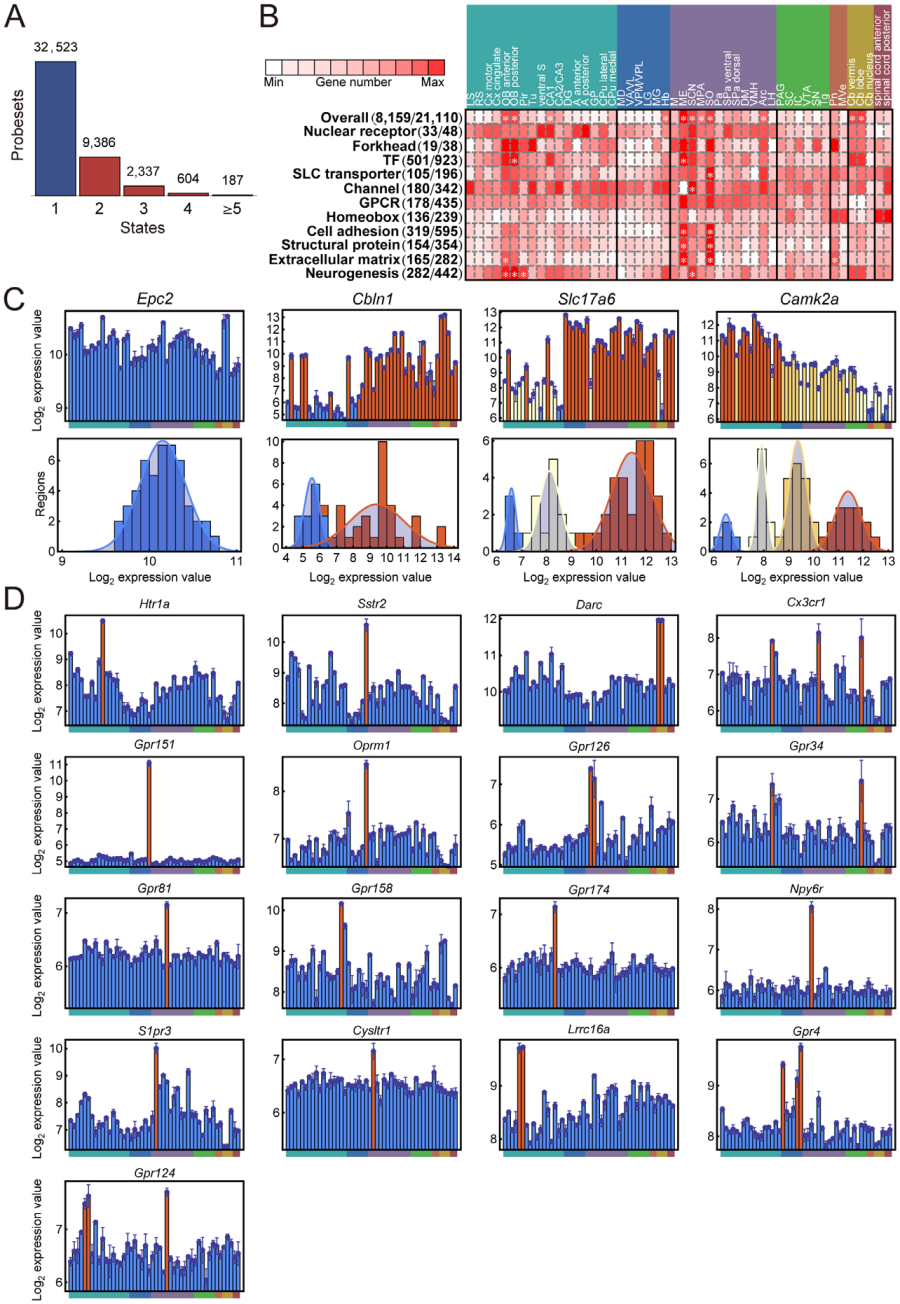
Multi-state genes. (**A**) Histogram giving the number of different states observed for the 45,037 non-control probe sets. (**B**) CNS regions that tended to be selected repeatedly as having “up” states of multi-state genes in various gene categories. Rows represent gene categories, and columns represent CNS regions. Asterisks indicate that the number of genes was significantly enriched in the designated CNS region (Bonferroni corrected *P*-value <0.05). (**C**) Examples of one-state, two-state, three-state, and four-state genes. Upper graphs show the expression values in 48 CNS regions, and lower graphs are histograms of the expression values and fitted Gaussian mixture models. The order of CNS regions in the expression graphs is the same as shown in **Figure 2B**. States are distinguished by color. (**D**) Examples of GPCR genes with multi-state spatial expression patterns. The order of CNS regions in the expression graph is the same as shown in **Figure 2B**.

“Multi-state” expression patterns, which are represented by a multi-modal distribution of gene expression, are thought to contribute to the spatial and temporal specificity of various biological functions [25], [26], [27]. To identify genes with such spatial expression patterns in the adult mouse CNS (“multi-state genes”), we used the BrainStars data from the 48 regions, and identified 8,159 genes (12,514 probe sets, ∼39% of all the genes in the DNA microarray) that showed multi-state expression patterns across the regions sampled (**Figure 2A**; see also **Text S1** and **Figure S2**). This multi-state expression analysis provides information about the “high” and “low” states of two-state genes, as well as the additional “middle” state(s) of genes that have three or more states. We noted that multiple states (“high,” “middle,” or “low” states) observed in the expression pattern could reflect either 1) different regulation of a gene's expression level in individual cells, or 2) regional heterogeneity, with different proportions of multiple cell types that express a given gene. Even in the latter case, classification as a multi-state gene indicates the existence of multiple states of a given gene in the CNS (because this is a preposition of regional heterogeneity). Thus, as the multi-state gene analysis may provide useful information for screening and detection of interesting genes with multi-state expression patterns, we decided to further analyze the states of multi-state genes.

Since different regions of the adult mammalian brain have specialized functions, the expression patterns of multi-state genes could reflect the roles played by the products (proteins) of these genes in carrying out such specific regional functions. For example, *cerebellin 1 precursor protein* (*Cbln1*), a two-state gene (**Figure 2C**), contributes to the control of synaptic structure and plasticity in the cerebellum [28], and *Solute carrier family 17 member 6* (*Slc17a6*), a three-state gene (**Figure 2C**), is a vesicular glutamate transporter in the thalamus [29]. We found that *Slc17a6* was highest in the thalamus and lowest in the cerebellum, and that in the hippocampus, which is telencephalic, it was expressed in the middle range (**Figure 2C**). *Calcium/calmodulin-dependent protein kinase II alpha* (*Camk2α*), a four-state gene (**Figure 2C**), is involved in long-term potentiation in the hippocampus [30]. We found that its highest expression level (“high” state) was in fact in regions of the hippocampus (cornu ammonis 1 [CA1], CA2/CA3, and dentate gyrus, DG). *Tyrosine hydroxylase* (*Th*), a rate-limiting enzyme for dopamine synthesis, was classified as a five-state gene, and its highest expression level (“high” state) was in the dopaminergic nuclei, ventral tegmental area (VTA), and SN.

We also found that some well-known GPCRs (G protein-coupled receptors) exhibited a two-state expression pattern in the BrainStars dataset (**Figure 2D**). For example, *5-hydroxytryptamine* (serotonin) *receptor 1A* (*Htr1a*) exhibited a two-state expression pattern with highest expression in the CA1 [31], *Somatostatin receptor 2* (*Sstr2*) in the habenular nucleus (Hb) [32], *Duffy blood group*, *chemokine receptor* (*Darc*) in the cerebellar cortex vermis (Cb vermis) and cerebellar cortex lobe (Cb lobe) [33], and *Chemokine (C-X3-C) receptor 1* (*Cx3cr1*) in the GP, supraoptic nucleus (SO) and SN [34]. In addition, we found further interesting examples; *G protein-coupled receptor 151* (*Gpr151*) exhibited a two-state expression pattern with the highest expression in the Hb (**Figure 2D**), which is consistent with the previous report that the expression of *Gpr151* is localized in the Hb of adult mouse brain [35]. *Gpr151* shows 25–26% identity and 41–43% similarity at the amino-acid level with the galanin-receptor subfamily, and is inferred to respond to Galanin. Since Galanin is related to pain [36] and Hb is also a nucleus related to pain, *Gpr151* might have a pain-related function. Interestingly, *opioid receptor*, *mu 1* (*Oprm1*), the opioid receptor related to analgesic effects of morphine, also exhibited a two-state expression pattern with the highest expression in the Hb (**Figure 2D**). Another example is the *G protein-coupled receptor 126* (*Gpr126*) showing a two-state expression pattern with high expression in the median eminence (ME) and suprachiasmatic nucleus (SCN) (**Figure 2D**). ME and SCN are centers for the photoperiodic calendar and circadian clock, respectively. Although *Gpr126* was recently reported to drive the differentiation of promyelinating Schwann cells in the peripheral nervous system (PNS) probably through elevating cAMP levels [37], the function of *Gpr126* in the CNS has not yet been clarified. One possible function of *Gpr126* in the CNS might be related to the photoperiodic calendar or circadian clock through the elevation of cAMP levels. The *G protein-coupled receptor 34* (*Gpr34*) was also highly expressed in the GP and SN. Although the function of *Gpr34* in the brain has not been clarified yet, it is known that SN and GP have a shared function in working as the output nuclei of the basal ganglia [38], and therefore, *Gpr34* might have a physiological function related to this process. We also found multi-state expression patterns of GPCRs that have not been yet examined so much in the adult mouse brain. For example, we noted that *G protein-coupled receptor 81* (*Gpr81*) was highly expressed in the SO, *G protein-coupled receptor 158* (*Gpr158*) in the caudate putamen lateral (CPu lateral), *G protein-coupled receptor 174* (*Gpr174*) in the GP, *neuropeptide Y receptor Y6* (*Npy6r*) in the SCN, and *sphingosine-1-phosphate receptor 3* (*S1pr3*) and *cysteinyl leukotriene receptor 1* (*Cysltr1*) in the ME (**Figure 2D**). We also found *Leucine rich repeat containing 16A* (*Lrrc16a*) expressed at high levels in the olfactory bulb anterior (OB anterior) and posterior (OB posterior), *G protein-coupled receptor 4* (*Gpr4*) in the mediodorsal thalamic nucleus (MD), medial geniculate nucleus (MG) and Hb, and *G protein-coupled receptor 124* (*Gpr124*) in the OB anterior, OB posterior and SO (**Figure 2D**).

Region-specific functions of multi-state genes were also evident at the level of individual gene categories (**Figure 2B**, and see also lists in BrainStars database, http://brainstars.org/). For example, 33 out of the 48 nuclear receptor genes in the DNA microarray were significantly overrepresented among the multi-state genes (Fisher's exact test, *P* = 0.013), supporting previous findings that these genes are differentially expressed in regions of the adult mouse brain [39], [40]. In addition, 136 of 239 homeobox genes, which control developmental processes in the embryo [41], were also significantly overrepresented among the multi-state genes (Fisher's exact test, *P* = 5.0×10^−4^), supporting previous and recent findings that suggest they play a role in the adult body [42], [43] and brain [11]. Finally, members of the cell adhesion and extracellular matrix gene categories were also overrepresented among the multi-state genes (Fisher's exact test, *P* = 5.0×10^−6^, and *P* = 4.1×10^−5^, respectively).

### Regional marker genes

**Figure 3:**
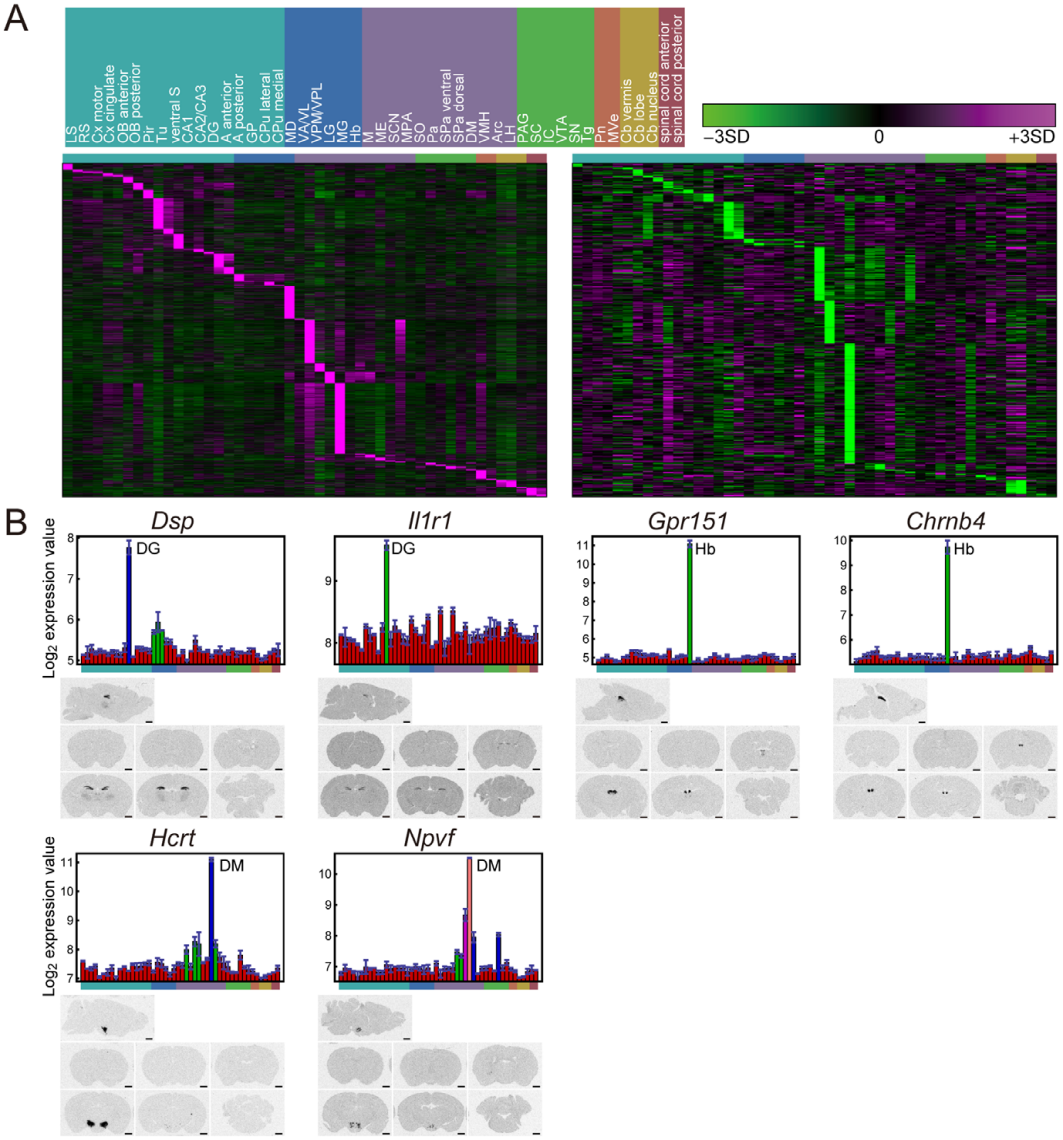
Regional marker gene candidates. (**A**) Heat maps of the expressions of marker gene candidates. Genes expressed at higher (left-lower) or lower (right-lower) levels in single regions are shown. The columns of the heat maps represent CNS regions whose order is shown at the left top of this panel. (**B**) GeneChip expression profiles (upper) of six marker gene candidates: *Dsp* (highly expressed in the DG), *Il1r1* (DG), *Gpr151* (Hb), *Chrnb4* (Hb), *Hcrt* (DM), and *Npvf* (DM) were also confirmed to be expressed in the corresponding regions by *in situ* hybridization (lower). Inferred states of marker gene candidates are distinguished by color in the upper charts. The order of CNS regions in the expression graphs is the same as in **Figure 2B**.

The data of multi-state expression patterns among CNS regions in the BrainStars dataset can be used to identify candidate genes whose expression levels can “mark” a specific CNS region. To find such candidate genes, we defined “marker” genes as the subclass of multi-state genes whose highest (or lowest) state of expression occurs in only a single CNS region. We found 2,573 (high) and 381 (low) probe sets (1,889 and 323 genes, respectively) for such marker genes (**Figure 3A** and see also lists in BrainStars database, http://brainstars.org/). For example, the highest expression of a multi-state gene *Gpr151* can “mark” Hb in the adult mouse brain (**Figure 3B**). We performed ISH with several of these marker gene candidates, and the results validated their regional specificity (**Figure 3B**). We noted that the marker gene candidates included genes related to a specialized function of each region. For example, *choline acetyltransferase* (*Chat*), a rate-limiting enzyme for acetylcholine synthesis, was a marker gene candidate for the Hb, a *cholinergic* basal forebrain complex. We also noted that among marker gene candidates expressed by a certain region, we sometimes identified gene pairs that constituted a transcriptional regulator and its target gene. For example, *nuclear receptor subfamily 0*, *group B*, *member 1* (*Nr0b1*) and *nuclear receptor subfamily 5*, *group A*, *member 1* (*Nr5a1*), marker gene candidates for the subparaventricular zone dorsal (SPa dorsal) region, are known to be co-expressed [44], and *Nr0b1* regulates *Nr5a1* [45].

### Internal control gene candidates in CNS

**Figure 4:**
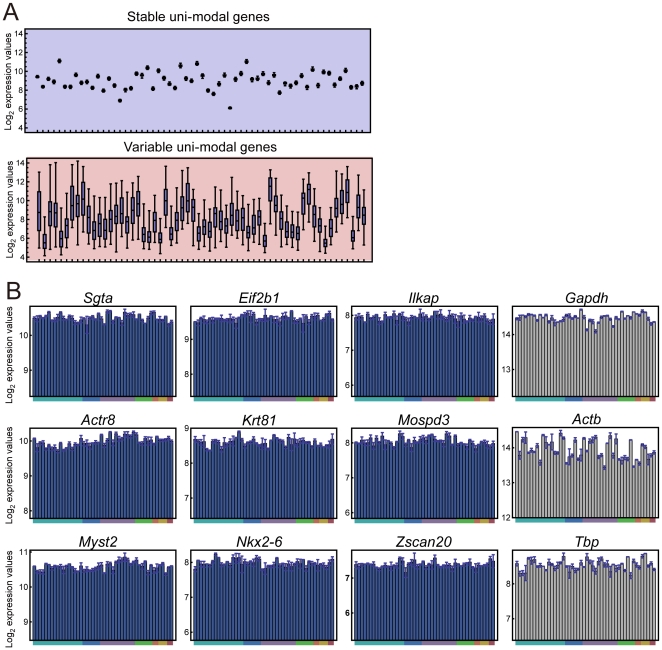
Internal control genes. (**A**) One-state genes with the 50 most stable (left panel) and variable (right panel) expression patterns. Each box shows the 0.25- to 0.75-quantiles of expression levels in 48 CNS regions for each probe set, and the error bars show the range of expression for all 48 regions. (**B**) Expression graphs for nine stable one-state genes identified in this study and three well-known internal control genes (*Gapdh*, *Actb*, and *Tbp*, right). The order of CNS regions in the expression graphs is the same as in **Figure 2B**. Top row, genes for metabolic process proteins. Middle row, genes for structural proteins. Bottom row, genes for transcription factors, expressed at high (left), intermediate (middle), and low (right) levels.

In addition to expression differences among the CNS regions discussed above, we also focused on a final class of genes which encompassed those that did not exhibit multi-state expression patterns in CNS regions. We called these “one-state” genes, because they were expressed unimodally across the CNS regions, and roughly followed a log-normal distribution (**Figure 2A and 2C**). Some one-state genes exhibited stable expression patterns, characterized by a log-normal distribution with small variance, whereas others exhibited a more variable expression, showing a log-normal distribution with larger variance (**Figure S3A**). By using a “variability score” determined from the expression data (**Materials and Methods** and **Figure S3B–D**), we identified 1,055 “stable” one-state genes (variability score less than −1.0) and 2,362 “variable” one-state genes (variability score more than 1.0) in the adult mouse CNS (**Figure 4A**, and see also lists in BrainStars database, http://brainstars.org/). We also confirmed the expression patterns of some of the stable and variable one-state genes by q-PCR (**Figure S3E–L**).

We observed that the stable and variable expression patterns of the one-state genes seemed to correlate with the subcellular localizations, molecular functions, and biological processes of their products (**Text S1** and **Figure S3M–T**). Although each functional category contained some members with stable or variable expression tendencies, remarkably stable one-state genes were found in most of the categories, including the metabolic process proteins, structural proteins, and transcription factors (**Figure 4B**). These genes could be novel candidates for internal controls in experiments using various methodologies, including q-PCR and ISH. They may prove to be more appropriate controls than the commonly used *glyceraldehyde-3-phosphate dehydrogenase* (*Gapdh*), *actin*, *beta* (*Actb*), or *TATA box binding protein* (*Tbp*), for some experiments (**Figure 4B**).

### Inferred connections among CNS regions

As one application of the multi-state expression analysis among CNS regions, we focused on genes related to ligands and receptors of neurohormones (NHs) and neurotransmitters (NTs). In the CNS, various NHs and NTs are secreted from neurons to convey information among distinct regions [46]. Therefore, expression data (especially multi-state expression patterns) for NH and NT (NH/NT) genes may be useful for investigating interconnections among CNS regions and intraconnections within the same CNS region.

**Figure 5:**
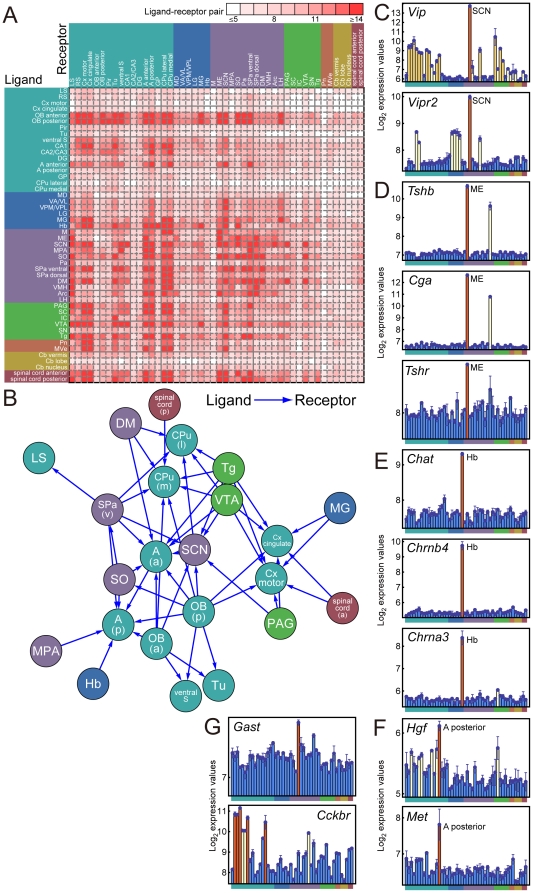
Inferred connections among CNS regions. (**A**) Pairs of CNS regions that tended to express the ligand gene for a neurohormone (NH) or neurotransmitter (NT) in one region and its cognate receptor gene in the other. The color of each tile represents the number of ligand-receptor pairs that had “up” states in the corresponding pair of CNS regions. (**B**) Graphical representation of the putative connections among CNS regions. Arrows originate in the ligand-expressing region and point to the region expressing the cognate NH/NT receptors, when >17 (0.05 quantile of the distribution of all combinations of regions) ligand–receptor pairs were expressed in the two regions. (**C**–**F**) Examples of inferred intrinsic ligand-receptor connections. (**G**) Examples of inferred extrinsic connections. The order of CNS regions in the expression graphs is as in **Figure 2B**.

To analyze the expression patterns of multi-state NH/NT genes, we first made a list that included the multi-state genes for the ligands themselves and those for enzymes that were rate-limiting in the biosynthesis of these ligands. Here we termed both of these categories as “ligand” genes. We also included the genes for NH/NT receptor proteins (i.e., “receptor” genes). Beginning with the multi-state NH/NT genes, we analyzed the ligand-receptor expressions in distinct CNS regions and found 68 neurohormone (NH) and neurotransmitter (NT) signaling pathways out of a total of 23,864 ligand-receptor interactions (including 519 intrinsic ligand-receptor interactions within the same CNS region) (see lists in BrainStars database, http://brainstars.org/). We counted the number of NH/NT ligand-receptor expressions for each CNS region pair, and drew a density plot of these counts (**Figure 5A**). In this density plot, we found strong connections between the hypothalamic and olfactory bulb regions. We have represented these findings in a network graph, which illustrates the presence of more than 17 ligand-receptor pairs (the 0.05 quantile of the distribution of all combinations of regions, and also with *P*<0.01 in a binomial test with Bonferroni correction) with arrows drawn between the CNS regions expressing ligand genes and those expressing their cognate receptors (**Figure 5B**). This analysis confirmed the findings from the density plot. Note that the network graph shows many arrows coming into the SCN, the circadian-clock center [47]. This implies that the SCN receives a wide variety of environmental or internal information from distinct CNS regions, allowing it to keep proper circadian timing. This finding is consistent with a previous report showing that many NH/NT pathways are active in the SCN [48].

We next analyzed the NH/NT ligand-receptor expressions within given CNS regions to infer their intrinsic connections. To identify such intraconnections, we retrieved pairs of ligand and receptor genes that had at least one common “high”- or “up”-state region (see lists in BrainStars database, http://brainstars.org/), and ranked them according to the total number of ligand-receptor matched states. One possible signaling pathway of intrinsic ligand-receptor interactions was the vasoactive intestinal peptide (VIP) signaling pathway (**Figure 5C**; the ligand gene was *Vip* and the receptor gene was *vasoactive intestinal peptide receptor 2* [*Vipr2*]). These two genes were highly expressed in the SCN, supporting previous findings that *Vip* and *Vipr2* contribute to the synchronization of clock cells within the SCN [49], [50], [51]. A second example of an intrinsic pathway was the thyroid-stimulating hormone (TSH) signaling pathway (**Figure 5D**; ligand genes were *glycoprotein hormones*, *alpha subunit* [*Cga*] and *thyroid stimulating hormone*, *beta subunit* [*Tshb*], and the receptor gene was *thyroid stimulating hormone receptor* [*Tshr*]). These three genes were highly expressed in the ME, a possible center for the photoperiodic calendar, supporting previous findings that the TSH signaling pathway is involved in photoperiodism [52], [53] and that that *Tshr* itself is required to maintain the high expression of *Tshb* in the ME [53]. A third example was the acetylcholine signaling pathway (ligand gene was *Chat* and receptor genes were *cholinergic receptor*, *nicotinic*, *alpha polypeptide 3* [*Chrna3*] and *cholinergic receptor*, *nicotinic*, *beta polypeptide 4* [*Chrnb4*]); these three genes were highly expressed in the Hb, one of the cholinergic nuclei (**Figure 5E**). A fourth example was the hepatocyte growth factor (HGF) signaling pathway (**Figure 5F**; ligand gene was *Hgf*, receptor gene was met proto-oncogene [*Met*]). These two genes were expressed in the amygdala posterior (A posterior). *Hgf* and *Met* are expressed in the brain [54], and their signaling mediates multiple neurodevelopmental and neurophysiological processes. However, little is known about the function of HGF in the amygdala. HGF infusion into the cerebral lateral ventricles influences anxiety in rats [55]. Because the amygdala has an important role in fear and anxiety [56], the HGF signaling pathway in the amygdala may be important for emotion. We also analyzed NH/NT ligand-receptor expressions among different CNS regions to infer extrinsic connections. To identify such interconnections, we retrieved pairs of ligand and receptor genes that had “up” states in different CNS regions. One example of a possible extrinsic ligand-receptor interaction was the Gastrin signaling pathway (**Figure 5G**; the ligand gene was *gastrin* [*Gast*] and the receptor gene was *cholecystokinin B receptor* [*Cckbr*]). *Gast* was expressed in the medial preoptic area (MPA), and *Cckbr* was mainly expressed in the retrosplenial cortex (RS), Cx motor, Cx cingulate, piriform cortex (Pir), and A posterior. The amygdala plays a key role in fear and anxiety [56], as mentioned above, and *Cckbr* knock-out mice are less anxious than normal mice [57], implying a possible role of Gastrin signaling between the MPA and the amygdala.

### Comparison of the BrainStars with other resources

**Figure 6:**
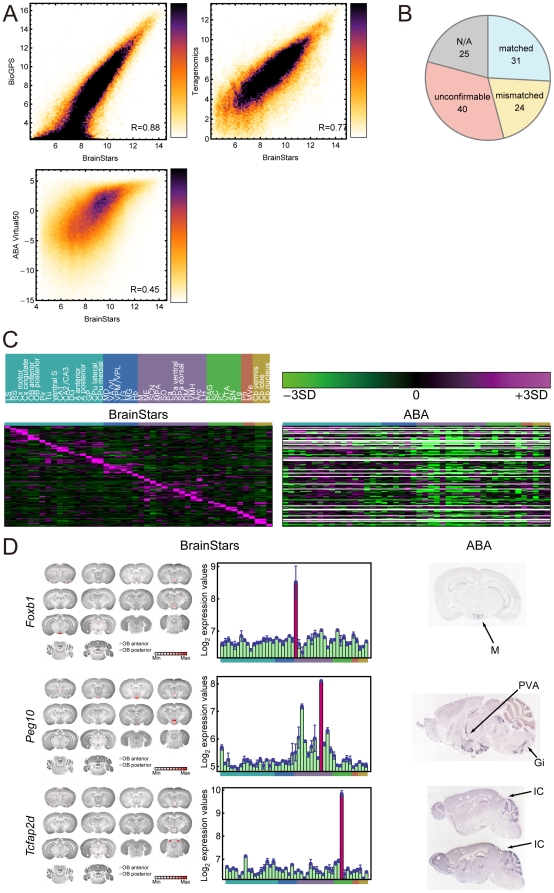
Comparison of BrainStars dataset with other resources. (**A**) Scatter plots comparing the BrainStars expression datasets with the BioGPS (top-left), Teragenomics (top-right), and Allen Brain Atlas (ABA) expression level (bottom-left) datasets. All expression values were log_2_-transformed. (**B**) Summary of the comparison of our marker gene candidates with ABA. Our 120 marker gene candidates with corresponding entries in the target dataset were classified as “matched”, “mismatched”, “unconfirmable”, or “N/A (not available)”. (**C**) Heatmaps of 120 marker gene candidates in the BrainStars, and ABA datasets. (**D**) The BrainStars, and ABA expression data for three marker gene candidates: *Foxb1*, *Peg10*, and *Tcfap2d*, show, respectively, agreement in datasets, or a lack of agreement between the datasets. For each gene, the BrainStars expression values were mapped onto slice images and are also represented as bar graphs, and the ABA *in situ* images are shown. The order of CNS regions in the expression graphs is as in **Figure 6C**. PVA: paraventricular thalamic nucleus, anterior part. Gi: gigantocellular reticular nucleus.

Although the BrainStars dataset is intended to be a valuable resource in itself, it is probably most useful when compared and combined with other available datasets that show gene expression in the adult mouse brain, such as BioGPS [58], Teragenomics [11], and the Allen Brain Atlas (ABA) [9]. To compare these datasets, we first evaluated the global correlation between the BrainStars expression dataset and the other resources (BioGPS, Teragenomics, and ABA). The Pearson's correlation coefficient between the BrainStars dataset and the BioGPS dataset was 0.88 (**Figure 6A** **top-left**), and the correlation between the BrainStars dataset and the Teragenomics dataset was 0.77 (**Figure 6A** **top-right**). The correlation between the BrainStars dataset and the ABA dataset (“expression energy,” see Lau *et al* [59] for its definition) was 0.45 (**Table S2**; **Figure 6A** **bottom-left**). These results were similar to a published comparison among the GNF SymAtlas (BioGPS), Teragnomics, and ABA datasets [21], in which the Pearson's correlations between the GNF SymAtlas (BioGPS) and Teragnomics datasets were 0.71–0.73, and between ABA and each of the other datasets were 0.39–0.52. These results suggest that the correlation level between the BrainStars dataset and the other datasets is acceptable, even though our sampling areas were much smaller than those used for the GNF SymAtlas (BioGPS) and Teragnomics datasets. We also found high variability in the correlations between our expression dataset and the ABA dataset (0.32–0.56) among the sampled regions (**Table S2**). The variability in the correlations between the BrainStars and ABA datasets depended to some extent on the complexity of the sampled region: the hippocampus and cortex, which are large and homogeneous, showed higher correlations, and the hypothalamic regions, which are smaller and more complex, showed lower ones.

We next evaluated our marker gene candidates with the ABA dataset at the level of individual genes (**Table S3**; **Figure 6B and 6C**). Of the 120 marker gene candidates determined in the BrainStar dataset (see **Materials and Methods**), 95 could be associated with corresponding entries in the ABA dataset (**Figure S4**). Of the 95 marker gene candidates associated with the ABA dataset, 31 were confirmed in the same regions (“matched”), 24 were discrepant (“mismatched”), and 40 could not be confirmed due to a lack of good-quality expression data in the ABA dataset (“unconfirmable”). Examples of marker gene candidates that were matched (*forkhead box B1* [*Foxb1*]) and mismatched (*paternally expressed 10* [*Peg10*], and *transcription factor AP-2*, *delta* [*Tcfap2d*]) are shown in **Figure 6D**. *Peg10*, which was expressed only in the DM in the BrainStars dataset, was also expressed in the PVA (paraventricular thalamic nucleus, anterior part) and the Gi (gigantocellular reticular nucleus) in the ABA dataset. *Tcfap2d* could not be detected only in the ABA dataset, because of non-specific signals in the ISH images.

Among the 24 “mismatched” genes in the comparison with the ABA dataset, we also found several that were expressed in additional regions in the ABA dataset (e.g., our Hb marker gene candidate *Gpr151* was also expressed in the PVA, and our MD marker gene candidate *gastrulation brain homeobox 2* [*Gbx2*] was also expressed in the PVA). Furthermore, the expressions of the remaining genes were detected in different regions from our dataset (e.g., our A posterior marker gene candidate *Met* was expressed in the lateral septal nucleus [LS] in the ABA dataset). Among the genes that were “unconfirmable” by comparison with the ABA dataset, we found that our ME marker gene candidates were not detected in the ABA dataset, which might have been caused by a disproportionate loss of the ME during slice dissection. We also found cases in which signals that were significant in the BrainStar dataset were not detected in the ABA dataset, owing to non-specific signals (e.g. for our LS marker gene candidate *PR domain containing 16* [*Prdm16*], and inferior colliculus [IC] marker gene candidate *Tcfap2d*) or to the narrow dynamic range of expression signals detected in the ABA dataset (e.g., for our SCN marker gene candidate *myocilin* [*Myoc*] and ventromedial hypothalamic nucleus [VMH] marker gene candidate *G protein-coupled receptor 103* [*Gpr103*]; see also **Text S1** and **Figure S5**).

## Discussion

In this study, we constructed a quantitative expression profile (expression map) of the adult mouse brain at the nucleus-level resolution. Our resource is especially useful for functional analyses focusing on specific functional brain regions. Marker gene candidates can be used to highlight CNS regions of interest. Multi-state genes can provide information for screening genes whose expressions in targeted regions are different from ones in other regions. One-state stable genes are novel internal control gene candidates for studies on the mouse brains. Although these kinds of data might be obtained with ISH resources (e.g. ABA), such data would be indirect and would require post-processing of ISH images, possibly introducing artifacts by quantification, image alignment, etc.

As the first set of analyses in the BrainStars project, we sampled 51 regions with distinct functions in the CNS of the adult mouse. We intended to include as many nuclei, loci, ganglia, and substantia as possible in the telencephalon, thalamus, hypothalamus, mesencephalon, and metencephalon. However, in this first analysis, we did not include some prominent brain regions, such as the SVZ (sub-ventricular zone; neurogenesis), LC (locus coeruleus; noradrenergic), raphe nuclei (serotonergic), and TMN (tuberomammillary nucleus, which is histaminergic), which will be sampled, analyzed, and reported in a future paper.

In collecting RNA of CNS regions, we used cylindrical punch samples, 0.5-mm thick and 0.5 mm in diameter, from 51 distinct CNS regions. This is a natural extension of our previous study on DNA-microarray-based expression profiling of SCN [60] to a quantitative and spatial genome-wide expression study of distinct functional regions of the adult mouse brain. To ensure the accuracy of our spatial expression dataset, we avoided potential artifacts caused by the circadian regulation of gene expression, which affects 2–10% of all genes [61], by taking samples of these small brain regions every 4 hours, starting at ZT0 (Zeitgaber time 0; the time of lights on), for 24 hours (6 time-point samples for each region). This strategy allowed us to avoid artifacts caused by temporal differences in gene expression [60], and therefore, to focus on spatial differences. We chose this strategy because we wanted to concentrate on spatial differences among the expression profiles of small brain regions, and this facilitates the identification of candidate genes. Once candidate genes are selected, we can collect time-course samples for these genes; this is one of our future works.

In our analysis of the expression data, we identified “multi-state” expression patterns in CNS regions. In the multi-state genes, we can find well-known CNS-active genes (e.g. *Camk2α*, *Th*) and GPCRs (e.g. *Htr1a*, *Sstr2*), and this result shows feasibility of our analysis method with using variational Bayesian inference. Furthermore, we identified many examples of genes that have not yet been examined so much in the adult mouse brain (e.g. *Gpr81*, *Gpr158*). These genes might have some physiological functions in the corresponding CNS regions, and further studies of such genes could be expected. We also performed a statistical analysis on region-specific functions of multi-state genes at the level of individual gene categories, and showed that several gene categories (e.g. nuclear receptor and cell adhesion) were over-represented. Although we need to be careful about the observations in which mRNA levels do not necessarily correlate with protein expression levels [62], [63], this statistical analysis indicate that digitalized expression patterns of multi-state genes imply some functional insights into the mouse adult CNS regions.

In our analysis of “multi-state” genes, we simply fit each expression profile to Gaussian mixture models with 1 to 6 normal distributions and chose the one with the best fit based on the variational Bayesian inference. There were several points we noticed. First, the analysis result contains genes with many states (5 states of Tyrosine hydroxylase, for example), and a few only show small changes among the states. However, such small changes among states are sometimes difficult to capture with current microarray technologies and the limited number of samples that we used. Therefore, we classified the multiple states identified through variational Bayesian inference into “on/off” or “up/down” states, and used these re-classified states in our further analyses. Second, there were “one-state” genes that look like “multi-state” genes, and there were “multi-state” genes that look like “one-state” genes. In some cases, there is not an obvious difference between some of the one-state and multi-state genes. These are because the fitness of Gaussian mixture models with one and multiple normal distributions were similar. We used the variational Bayesian inference because this method can select a more appropriate Gaussian mixture model than other methods such as EM (Expectation Maximization) algorithm [64]. We thus believe that the misclassification between “one-state” and “multi-state” genes were lower than other methods.

For “one-state” genes, we should be careful when interpreting their data because these “one-state” expressions may be caused by technical factors, such as the dynamic range of the microarray probe, alternative splicing not detectable by the probe, outdated probe design, etc. Moreover, since our sample set does not cover all CNS regions, developmental stages, and conditions such as environmental stress and diet, we cannot observe the change in a particular set of circumstances. However, we believe that we can use the “one-state” gene set in various studies, such as for identifying candidate positive control genes.

We also identified marker gene candidates for various CNS regions from the multi-state genes, and validated some of them by *in situ*. These genes can be used to highlight specific regions in the adult mouse brain for a range of further studies. Furthermore, we found that the candidate marker gene set included transcriptional regulators and its target gene pairs. Thus, it is possible that certain information about transcriptional regulation can also be retrieved from the marker gene candidates. We noted that a set of marker gene candidates in our genome-wide and nucleus-level expression data include region-specific genes that were not detected in similar resources. For example, the Allen Brain Atlas has a genome-wide ISH data and provides “fine structure” dataset (equivalent to “marker gene” candidates in this study) on their web site (http://mouse.brain-map.org/). In the “fine structure” dataset, *Gpr151* (marker gene candidate in Hb) and *Vip* (marker gene candidate in SCN) cannot be detected, although their regional specificities seemed to be found in the corresponding ABA ISH images. This may be caused by difficulties in quantitative analysis with ISH data or by their data quality for positioning brain regions, implying the advantage of BrainStars dataset in the quantitativeness.

As one application of the multi-state expression analysis among CNS regions, we searched for genes related to ligands and receptors of neurohormones (NHs) and neurotransmitters (NTs). We found 68 neurohormone (NH) and neurotransmitter (NT) signaling pathways in a total of 23,864 ligand-receptor interactions, and we drew an inferred ligand-receptor interaction map of the CNS regions. Although these inferred interactions represent a “possibility map” (i.e. there is a possibility of connection between two regions because they selectively express the appropriate ligands and receptors), these ligand-receptor interaction candidates will provide interesting hypotheses for future studies in neuroscience. We expect various neuroscience studies would be advanced by the close investigation of such candidate sets by experts in the field.

The results of the comparison among our BrainStars dataset and other resources indicate the advantages of our strategy for constructing the BrainStars database, which improved the dynamic range of detection (compared with the ABA). Although some discrepancies in the “mismatched” results between our database and the ABA may be caused by the limited number and size of the samples used for the BrainStars dataset, this limitation can be compensated for by comparing and combining datasets, preventing this from becoming a critical deficit of the BrainStars dataset. These comparisons indicate that no single method devised to date can provide complete genome-wide expression data for the adult mouse brain that has 1) a large dynamic range, 2) high spatial resolution, and 3) coverage of the whole brain. Because of the limitations of each method, we believe that the complementary and cooperative usage of these genome-wide expression datasets is the most useful platform for further investigation of the structure and function of the adult mouse brain. Therefore, to make the best use of these datasets, we constructed an integrated database and viewer for them (BrainStars viewer). The BrainStars viewer is publicly available at http://brainstars.org/.

The elucidation of the regulatory mechanisms of the mammalian brain is still a challenging goal that requires a variety of resources, including CNS expression maps. Our new resource should help accelerate the functional analysis of the mammalian brain and the elucidation of its regulatory network systems.

## Materials and Methods

### Ethics Statement

This study was approved by the Animal Care and Use Committee, Kinki University School of Medicine, and carefully followed the Guide for the Care and Use of Laboratory Animals, Kinki University School of Medicine (approved without IDs). Mice were also carefully kept and handled according to the RIKEN Regulations for Animal Experiments (AH18-02-18).

### Nucleus-level sampling of CNS regions

Balb/c mice (all mice were male) purchased 5 weeks postpartum, were adapted under a standard 12-h light/dark cycle (LD) for 2 weeks, before samples were obtained under LD or constant darkness (DD) conditions, every 4 h over 1 day, starting at ZT0. Slices (0.5-mm thick) of mouse brain were cut on a Mouse Brain Matrix (Neuroscience, Tokyo), frozen, and the specific regions were punched out bilaterally with a microdissecting needle (gauge 0.5 mm) under a stereomicroscope. We sampled 5–25 mice for each CNS region at each time point, and, as a result, samples from 30–150 mice were collected for each replicate of a single CNS region. This whole procedure was repeated twice (n = 2) to obtain experimental replicates for every CNS region.

### Microarray Analysis

The total RNA was prepared from the pooled samples for each region taken at all time points using Trizol reagent (Gibco BRL). The cDNA synthesis and cRNA labeling reactions were performed as previously described [65]. Affymetrix high-density oligonucleotide arrays for *Mus musculus* (GeneChip Mouse Genome 430 2.0) were hybridized, stained, and washed according to the Expression Analysis Technical Manual (Affymetrix). The expression values were summarized by the RMA method [66]. The resulting expression values were used in all the subsequent analyses. All data is MIAME compliant and the GEO accession number for the microarray data deposited and reported in this paper is GSE16496.

### Quantitative PCR

Quantitative PCR was performed with the ABI Prism 7900 and SYBR Green Reagents (Applied Biosystems). The cDNAs were synthesized from 0.25 µg of total RNA using Superscript II reverse transcriptase (Invitrogen). Samples contained 1× SYBR Green Master Mix, 0.8 µM primers, and 1/40 synthesized cDNA in a 10 µl volume. The PCR conditions were as follows: 10 min at 95°C, then 45 cycles of 15 s at 94°C, 1 m at 59°C. The absolute cDNA abundance was calculated using a standard curve obtained from murine genomic DNAs. We used Tbp as the internal control.

### *In situ* hybridization (ISH)

Mice were deeply anesthetized with ether and intracardially perfused with 10 ml saline and 20 ml of a fixative containing 4% paraformaldehyde in 0.1 M phosphate buffer (PB), pH 7.4. Mouse brain samples were postfixed in the same fixative for 24 h at 4°C, soaked in PB containing 20% sucrose for 48 h, and finally stored frozen at −70°C. The ISH method was described in detail previously [67]. Serial coronal and sagittal sections (40-µm thick) of the mouse brain were made using a cryostat. Fragments of cDNA were obtained by PCR, and the products were then subcloned into the PGEM-T easy vector (Promega). Radiolabeled probes were generated using ^35^S-UTP (PerkinElmer) via a standard protocol for cRNA synthesis. The primers used in the ISH were ctcacagtgatgctgctaagc (*Gpr151*, forward), ccctctgtctcttggccttc (*Gpr151*, reverse), ctacccagcgtgttatgggg (*Chrnb4*, forward), catgggagtagatctctgcc (*Chrnb4*, reverse), cgaggtctggagactactac (*Dsp*, forward), agcagaaccctcaacctctc (*Dsp*, reverse), gggagaaatgtcgctggat (*Il1r1*, forward), cataagggcacacaagacttcc (*Il1r1*, reverse), ctgagaggaatcccaaaagg (*Rfrp* [*Npvf*], forward), gctttccaccaggactctga (*Rfrp* [*Npvf*], reverse), ctgctgctgctgctactgct (*Hcrt*, forward), and gacgattctctgttggtgtgac (*Hcrt*, reverse).

### Hierarchical clustering of CNS regions with their expression profiles

For the hierarchical clustering of the 51 CNS regions and 102 samples, the correlation dissimilarity, i.e., 1-(Pearson's correlation coefficient) and Euclidean distance were used as distance functions, and a complete linkage method was used to build the clusters. For the statistical analysis of the separation of 51 CNS regions, pvclust clustering [68] was also performed with the four distance metrics indicated above. For every hierarchical clustering analysis, the natural expression values of 45,037 non-control probe sets in the 51 CNS regions or 102 samples were used.

### Gene categories

Genes for transcription factors (TF), channels, GPCRs, cell adhesion proteins, structural proteins, extracellular matrix proteins, and neurogenesis-associated proteins were retrieved using the corresponding gene ontology term assignments (GO:0003700 [transcription factor activity]/GO:0016563 [transcription activator activity]/GO:0016564 [transcription repressor activity], GO:0015267 [channel activity], GO:0004930 [G-protein coupled receptor activity], GO:0007155 [cell adhesion], GO:0005198 [structural molecule activity], GO:0031012 [extracellular matrix], GO:0022008 [neurogenesis], respectively), which were found in the annotation file (Mouse 430 2.0, na27) provided at the Affymetrix website. Genes assigned to GO:0003735 [structural constituent of ribosome] were excluded from the structural proteins. We chose 293 homeobox genes and 49 nuclear receptors, respectively, using the following references [40], [69]. NH/NT ligand/receptor genes, SLC transporter genes, and forkhead genes were manually retrieved. For the NH/NTs, the ligand-coding genes or the rate-determining enzymes for their biosynthesis were selected as “ligand genes,” and their receptors were selected as “receptor genes” for ∼140 neurohormones. SLC transporter genes and forkhead genes were also manually retrieved from the NCBI Entrez Gene database. All the gene category lists can be found in the BrainStars database (http://brainstars.org/).

### Identification of multi-state genes

Genes with multi-state expression patterns were identified with a variational Bayesian inference to fit a Gaussian mixture model [64]. We used the Gaussian mixture model with six components, in which each component has three parameters (mean, variance, mixture probability) with five hyper-parameters, and we assumed that components of Gaussian mixture have different variances. The detailed procedure for determining prior hyper-parameters and fitting Gaussian mixture models is described in the **Text S1** and **Figure S2**. We used log_2_-transformed expression values for the 45,037 non-control probe sets in the 48 CNS regions that did not include the retina, pituitary, or pineal. After the fitting procedure, the CNS regions were grouped according to predicted states. For example, CNS regions were classified into three groups for three-state genes because there were three states (“high,” “low,” and “middle”).

### Statistical analysis on multi-state genes

To test whether a multi-state gene with an “up” state was significantly enriched in a particular CNS region, we performed one-sided binomial tests to calculate its *P*-value based on the probability where a multi-state gene has an “up” state at a single CNS region: the total number of regions with “up” states of all unique multi-state genes/(the number of unique multi-state genes×the number of CNS regions), i.e., 117,097/(8,159×48). After the *P*-values were calculated, they were subjected to the Bonferroni correction.

To determine what kinds of genes were enriched among those repeatedly selected as having “up” states in pairs of regions, we performed the hyperGTest in the Bioconductor packages [24], which assigns *P*-values to show that a gene category (gene ontology, or GO term) is enriched in those genes repeatedly selected as having an “up” state in pairs of regions against all multi-state genes. After the *P*-values were assigned, false discovery rates (*FDR*s) were calculated within the GO molecular function, biological process, and cellular component classes for each pair of regions, and the GO terms whose FDR was less than or equal to 0.01 were retrieved.

### Identification of regional marker gene candidates

Multi-state genes whose expression levels were higher or lower in a single CNS region (marker gene candidates) than in the others were chosen as marker gene candidates. A multi-state gene was selected as a marker gene candidate of a designated CNS region if its probe set had only the single CNS region for the highest (or lowest) state of its expression.

### Expression variability analysis

We used 32,523 one-state probe sets. To assign variability scores to the chosen probe sets, we first filtered out probe sets that were not “present” in any CNS region. We regarded a probe set as “present” in a CNS region when both samples for the region were called “present” by the Affymetrix MAS 5.0 detection algorithm (Statistical Algorithms Description Document; http://www.affymetrix.com/support/technical/whitepapers/sadd_whitepaper.pdf). Of the 32,523 probe sets, 13,619 were filtered out. Next, we made a scatter plot of the mean (X-axis) and standard deviation (Y-axis) of the log_2_-transformed expression values for each of the “present” probe sets, and drew a curve showing the running median of the standard deviation (**Figure S3C**). Variability scores were calculated by dividing the standard deviation by its running median, and then applying log_2_-transformation (**Figure S3D**).

### Inferred connections among CNS regions

To analyze the expression patterns of the NH/NT genes, we first made a list that included the genes for the ligands themselves and those for enzymes that were rate-limiting in the biosynthesis of these ligands (“ligand” genes). We also included the genes for NH/NT receptor proteins (i.e., “receptor” genes). The list of NH/NT-related genes contained 176 that encoded ligands and 270 that encoded receptors, which were components of 118 NH and NT pathways: 6 for monoamines and acetylcholine, 4 for amino acids, 95 for peptides, 2 for gases, and 11 for other types of pathways. Multi-state genes comprised 253 of these NH and NT genes. For every CNS region pair, we then counted the number of NH/NTs whose ligand gene had an “up” state in one of the CNS region pair, and whose receptor gene was “up” state in the other region. In this analysis, we ignored the strength of expression, i.e., differences between the “middle” and “high” (highest-level) states.

We illustrated the presence of more than 17 ligand-receptor pairs that correspond to the 0.05 quantile of the distribution of all combinations of regions in **Figure 5B**. To test the significance of this cutoff number (i.e. 17) of NH/NTs used for illustrating the inferred network of CNS regions, we performed the bionomial test to calculate its *P*-value as follows. The number of unique multi-state genes was 8,159, and the total number of regions with “up” states of all the unique multi-state genes was 117,097. Thus, the probability that a CNS region pair was randomly chosen as a ligand-receptor pair region was (117,097/(8159×48))^2^. The number of NT/NHs whose ligand and receptor genes were multi-state genes was 68. We performed binomial tests with these parameters to estimate the *P*-values of the null hypothesis that the number of NT/NHs (for each of 0–68) was random, and adjusted the *P*-values with the Bonferroni correction.

### Comparison of datasets

To evaluate and compare the BrainStars dataset with the Allen Brain Atlas (ABA) datasets, we retrieved the expression values from the ABA dataset for brain regions that were sampled for the BrainStars database. We mapped our brain regions to the ABA dataset, and retrieved the mean expression values (defined as “expression energies” [59]), which are available from http://www.brain-map.org/. If no expression energy for a gene was found in a specific brain region, that region was excluded from the analysis. To compare globally the BrainStars dataset with the ABA dataset, we used our brain regions which excluded the retina, pituitary, pineal, and spinal cord, drew a scatter plot, and calculated Pearson's correlation coefficients. For each gene, the single probe sets (BrainStars) with the largest mean expression values were chosen. To compare globally the BrainStars and BioGPS datasets, the means of {Cx motor, Cx cingulate}, {A anterior, A posterior}, {CA1, CA2/CA3, DG}, {OB anterior, OB posterior}, {spinal cord anterior, spinal cord posterior}, {M, ME, SCN, MPA, SO, Pa, SPa ventral, SPa dorsal, DM, VMH, Arc, LH}, {Cb vermis, Cb lobe, Cb nucleus}, and {CPu lateral, CPu medial} in the BrainStars dataset were compared with the cerebral cortex, amygdala, hippocampus, olfactory bulb, spinal cord, hypothalamus, cerebellum, and dorsal striatum in the BioGPS dataset, respectively. To compare globally the BrainStars and Teragenomics datasets, the means of {A anterior, A posterior}, {CA1}, {CA2/CA3}, {Cb vermis, Cb lobe, Cb nucleus}, {Cx motor, Cx cingulate}, {DG}, {CA1, CA2 CA3, DG, ventral S}, {M, ME, SCN, MPA, SO, Pa, SPa ventral, SPa dorsal, DM, VMH, Arc, LH}, {IC}, {PAG, SC, IC, VTA, SN, Tg, MD, VA/VL, VPM/VPL, LG, MG, Hb}, {Cx motor}, {OB anterior, OB posterior}, {PAG}, {Pituitary}, {Pn, MVe}, {Retina}, {spinal cord anterior, spinal cord posterior}, {CPu lateral, CPu medial}, {SC} in the BrainStars dataset were compared with the amygdala, CA1, CA3, cerebellum, cerebral cortex, dentate gyrus, hippocampal formation, hypothalamus, inferior colliculus, “midbrain and diencephalon, no hypothalamus”, motor cortex, olfactory bulb, periaqueductal gray, pituitary, pons, retina, spinal cord, striatum, superior colliculus in the Teragenomics dataset, respectively.

To perform a more detailed comparison of the BrainStars dataset with the ABA dataset, we first selected up to three probe sets of BrainStars marker gene candidates that showed the largest expression changes within the CNS for each of 46 brain regions (without spinal cord anterior, spinal cord posterior, retina, pituitary and pineal), choosing 120 unique marker gene candidates (**Table S3**). We then manually compared these marker gene candidates against the ABA dataset, and classified them as “matched” when their regional expression was in the same location in the Allen Brain Atlas dataset, “mismatched,” when the genes were confirmed as not being marker genes in the corresponding brain region, or “unconfirmable” when the genes could not be confirmed due to a lack of good comparative data.

## Supporting Information

Figure S1 **Sampled adult mouse CNS regions.** (**A–E**) Hierarchical clustering of brain regions and samples with various distance metrics. Brain samples were clustered by (**A**) correlation dissimilarity, and (**B**) Euclidean distance. Brain regions were clustered by (**C**) Euclidean distance. Brain regions were also statistically clustered by (**D**) correlation dissimilarity and (**E**) Euclidean distance with significance scores (red and green scores). (**F**) Scatter plot comparing the experimental replicates of all CNS regions. X- and Y-axes show the log_2_-transformed expression value of each experimental replicates. (**G**) Correlation coefficients indicating the reproducibility of the experimental replicates of each CNS region.
(PDF)

Figure S2 **Multi-state genes.** (**A–C**) Determination of prior distributions by variational Bayesian inference of Gaussian mixture. (**A**) Distribution of the inverse of the median error variance. The median error variance was calculated as the median variance of the duplicated expression values (n = 2) in 48 brain regions for each probe set. (**B**) Plot of false-positive rates and false-negative rates generated by changing the α_0_ prior hyper-parameter. The X- and Y-axes show the false-negative rate ( = 1 – sensitivity) and 1 – the false-positive rate ( = specificity), respectively. Each curve represents a different false discovery rate (*FDR*) cut-off for the marker gene candidates, which was regarded as the true set for parameter evaluation. Gray lines show *y* = *x*+(constant), which represent equal sums of the false-positive and false-negative rates. (**C**) An example in which two mixture components overlapped and one state was nested into another state. A histogram of its expression values is shown.
(PDF)

Figure S3 **One-state genes.** (**A**) Distribution of the standard deviation of one-state (blue) and multi-state (red) genes. (**B**) Distribution of variability scores. The higher and lower variability scores of genes indicated that their expression levels were variable and stable, respectively. (**C**) Scatter plot of the standard deviations against the means of the log_2_-transformed expression values. Blue dots represent single probe sets, and the red curve shows their running median. (**D**) Scatter plot of the mean of the log_2_-transformed expression values and the variability scores. (**E–L**) Confirmation of several stable and variable one-state genes by q-PCR. The expression values relative to the *Tbp* expression are shown. Stable one-state genes *Sgta* (**F**) and *Egln2* (**H**) were also stable by q-PCR (**E** and **G**, respectively), whereas the variable one-state genes *Kcnab1* (**J**) and *Nrip3* (**L**) were also variable by q-PCR (**I** and **K**, respectively). (**M–T**) Correlation of the variability score with the subcellular localization (**M**–**P**), molecular function (**Q**, **R**), and biological process (**S**, **T**) of the gene products. Each graph represents the ratios of genes associated with the Gene Ontology term for 25 subsets of one-state genes, sorted by the rank of their variability scores. The false discovery rate (*FDR*) for the enrichment of gene functions in stable or variable genes are also shown.
(PDF)

Figure S4 **Comparison of BrainStars dataset with other resources.** Candidates for 120 marker genes from the BrainStars dataset are shown along with results from the Allen Brain Atlas (ABA) dataset. For each gene, the BrainStars expression values were mapped onto images of brain slices (upper-left) and represented in a bar chart (upper-right), and the ABA expression values (“expression energies”) from a coronal cross-section at the expressing CNS region are shown (lower-left).
(PDF)

Figure S5 **Comparison of reproducibility and quantitativeness between BrainStars and ABA datasets.** (**A–B**) Scatter plot showing the reproducibility of the experimental replicates of all the CNS regions in the BrainStars (**A**) and Allen Brain Atlas (**B**) projects. Blue lines indicate 2-fold changes. (**C–D**) The proportion of replicated data points showing a difference within 2-fold in the BrainStars (**C**) and Allen Brain Atlas (**D**) projects. The dynamic range, which we defined as the range of more than 50% of replicated data points that showed a less-than 2-fold change, was from 2^4^ to 2^14^ (∼10^3.0^-fold) for the BrainStars project and 2^2^ to 2^5^ (∼10^0.9^-fold) for the ABA project. (**E–F**) Correlation coefficient showing the reproducibility of experimental replicates of each CNS region in the BrainStars (**E**) and Allen Brain Atlas (**F**) projects. (**G**) *Myl4* expression at 51 CNS regions in our data. Error bars show standard errors. In the Cx motor and Cx cingulate, the *Myl4* expressions were greatly changed with small standard errors. (**H**) Number of redundant probes for the same transcript in each database. (**I–J**) Maximum correlation coefficient between redundant probes for the same transcript. (**I**) Distribution of the maximum correlation coefficients for the BrainStars oligo-probes. (**J**) Distribution of the maximum correlation coefficients in the Allen Brain Atlas.
(PDF)

Table S1 **Sample information for all 51 central nervous system (CNS) regions.**
(DOC)

Table S2 **Pearson's correlation coefficients of the BrainStars dataset with the Allen Brain Atlas (ABA) dataset.**
(DOC)

Table S3 **Comparison of the marker gene candidates in the BrainStars dataset with the Allen Brain Atlas (ABA) dataset.**
(DOC)

Text S1 **Supporting materials and methods, and results.**
(DOC)
