# Supplementary figures and images for: Quantitative Expression Profile of Distinct Functional Regions in the Adult Mouse Brain
Source: PLoS One. 2011 Aug 12;6(8):e23228. doi: 10.1371/journal.pone.0023228 (PMC3155528; doi:10.1371/journal.pone.0023228)

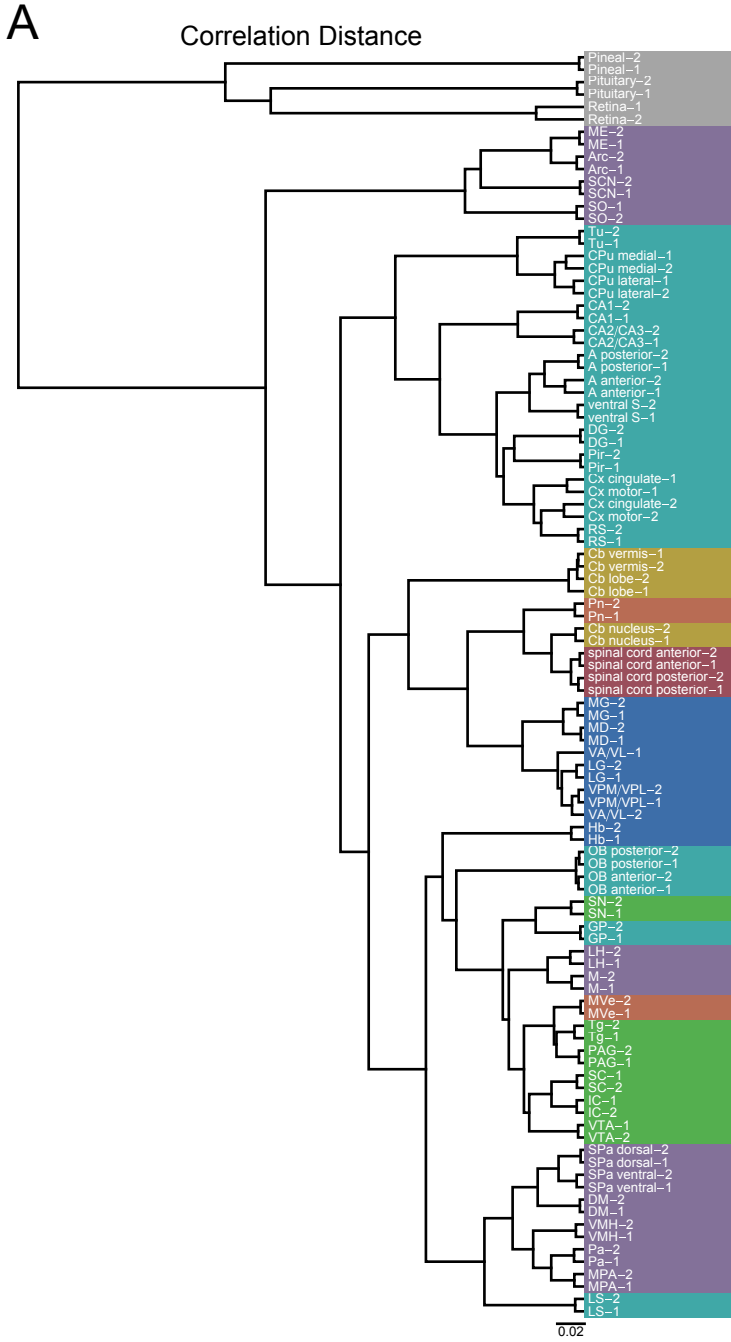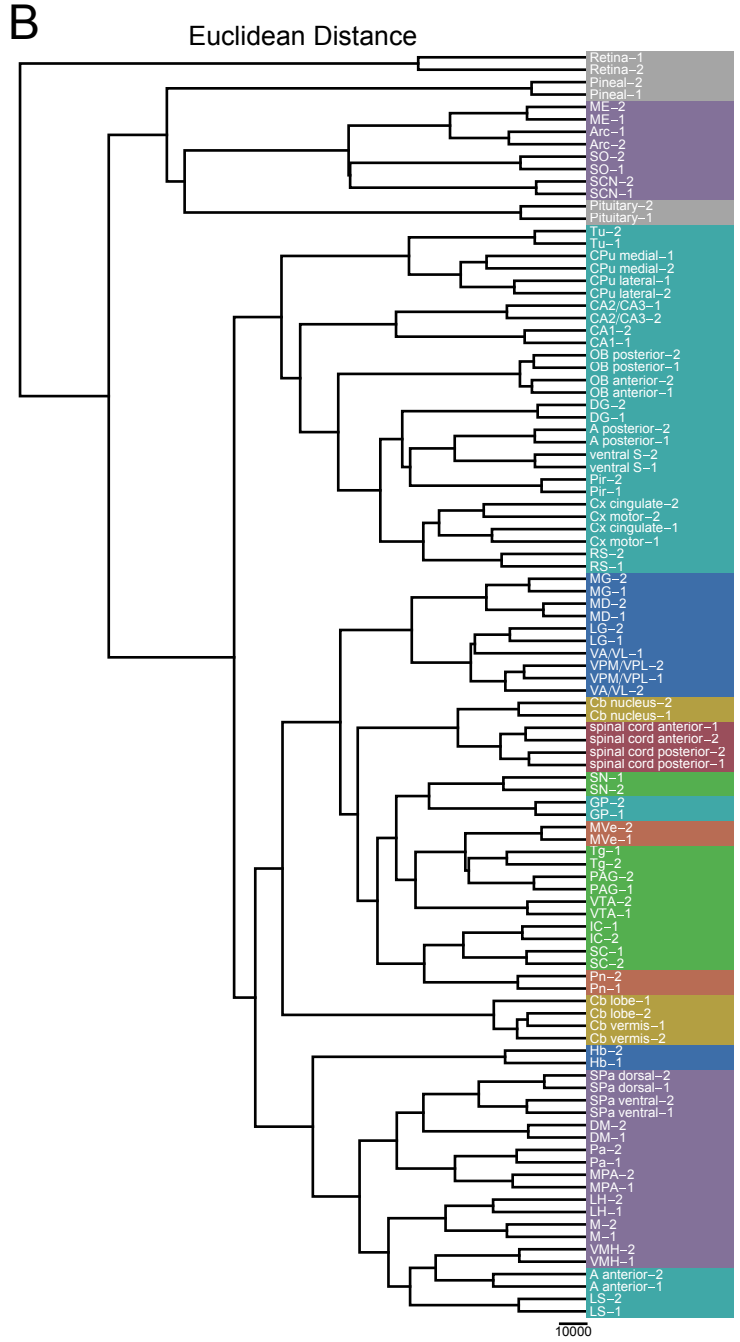

Figure S1 (1/3)  
Kasukawa *et al.*

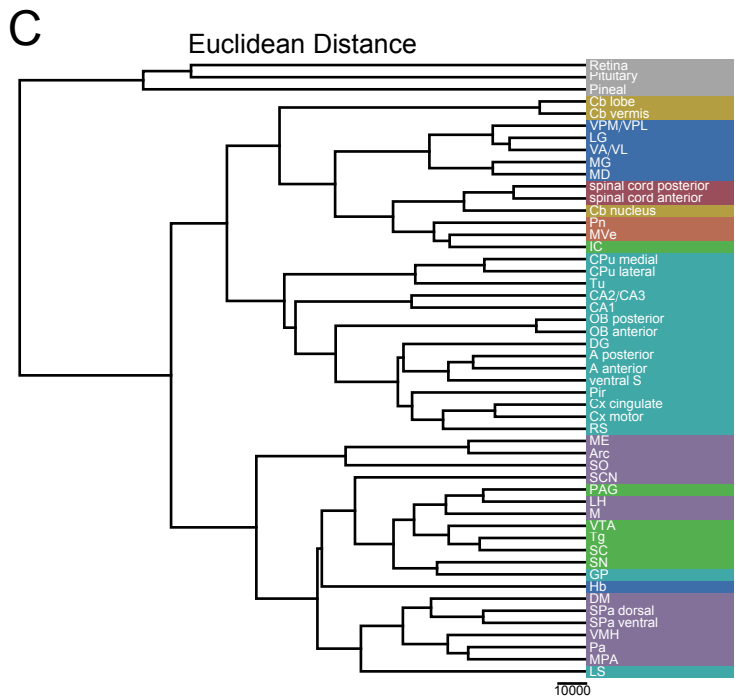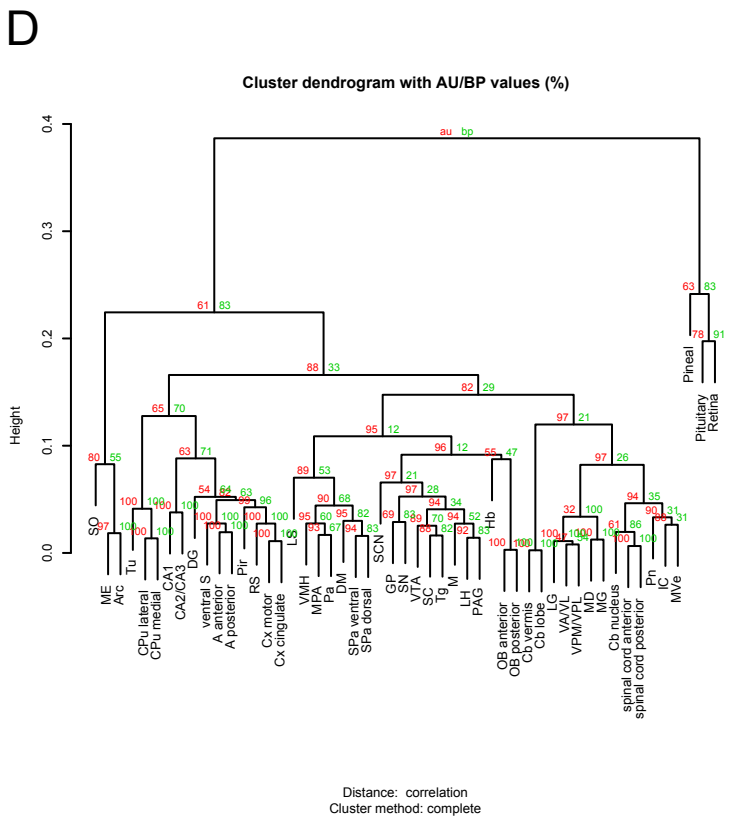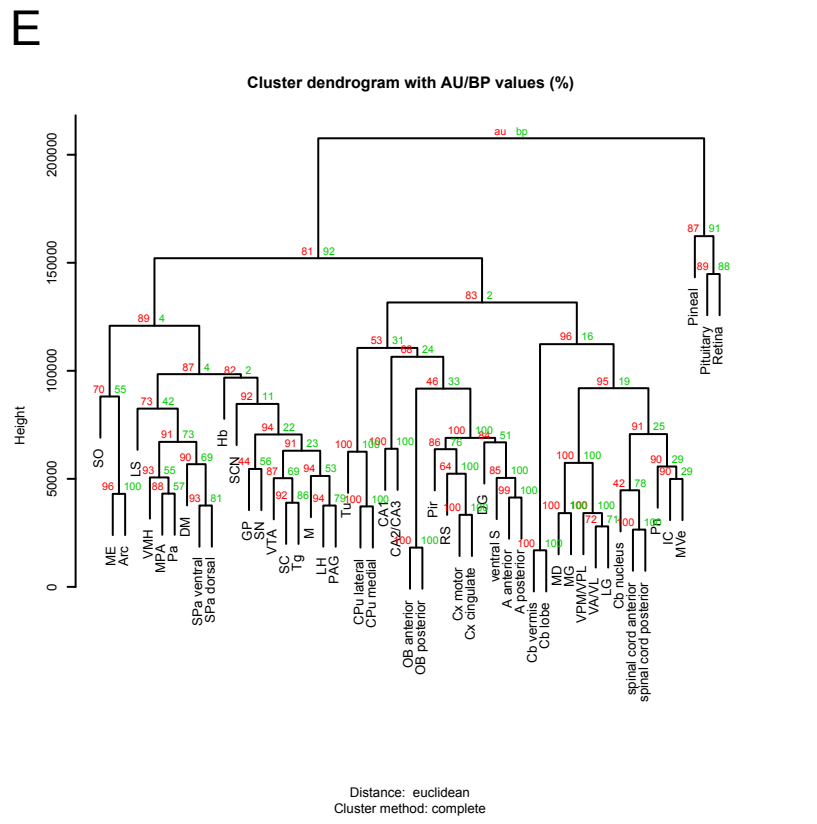

Figure S1(2/3)  
Kasukawa *et al.*

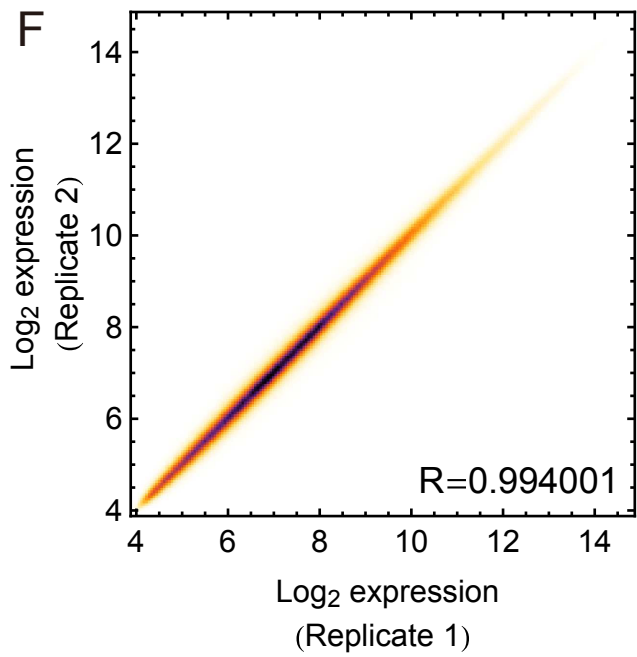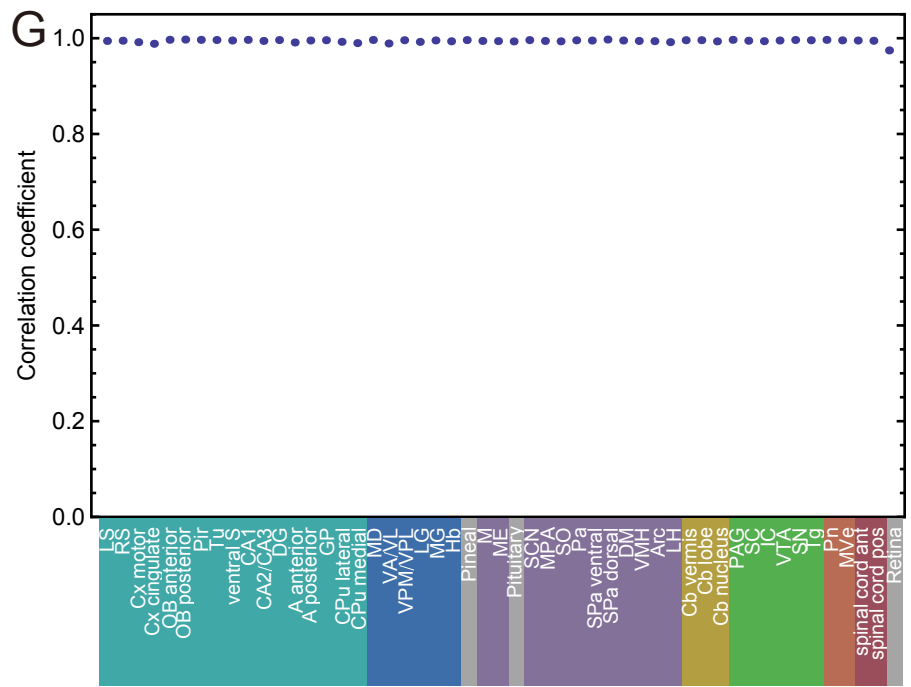

Figure S1 (3/3)  
Kasukawa *et al.*

Supplement: Figure S1 — Sampled adult mouse CNS regions. (A–E) Hierarchical clustering of brain regions and samples with various distance metrics. Brain samples were clustered by (A) correlation dissimilarity, and (B) Euclidean distance. Brain regions were clustered by (C) Euclidean distance. Brain regions were also statistically clustered by (D) correlation dissimilarity and (E) Euclidean distance with significance scores (red and green scores). (F) Scatter plot comparing the experimental replicates of all CNS regions. X- and Y-axes show the log2-transformed expression value of each experimental replicates. (G) Correlation coefficients indicating the reproducibility of the experimental replicates of each CNS region. (PDF) [file pone.0023228.s001.pdf]

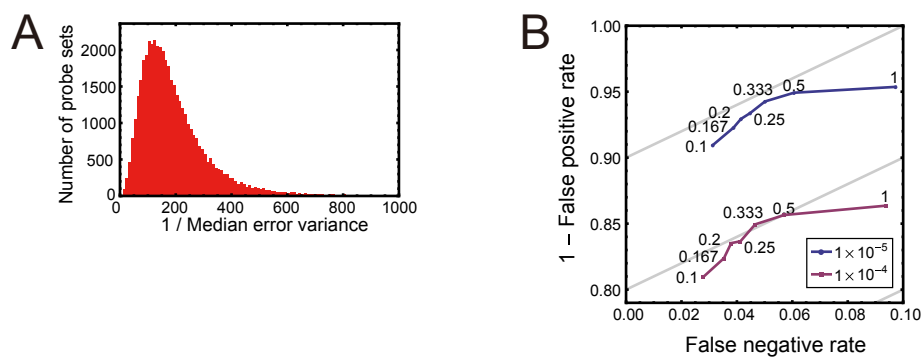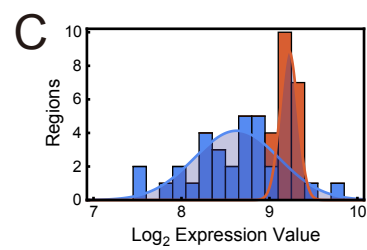

Figure S2 Kasukawa *et al.*

Supplement: Figure S2 — Multi-state genes. (A–C) Determination of prior distributions by variational Bayesian inference of Gaussian mixture. (A) Distribution of the inverse of the median error variance. The median error variance was calculated as the median variance of the duplicated expression values (n = 2) in 48 brain regions for each probe set. (B) Plot of false-positive rates and false-negative rates generated by changing the α0 prior hyper-parameter. The X- and Y-axes show the false-negative rate ( = 1 – sensitivity) and 1 – the false-positive rate ( = specificity), respectively. Each curve represents a different false discovery rate (FDR) cut-off for the marker gene candidates, which was regarded as the true set for parameter evaluation. Gray lines show y = x+(constant), which represent equal sums of the false-positive and false-negative rates. (C) An example in which two mixture components overlapped and one state was nested into another state. A histogram of its expression values is shown. (PDF) [file pone.0023228.s002.pdf]

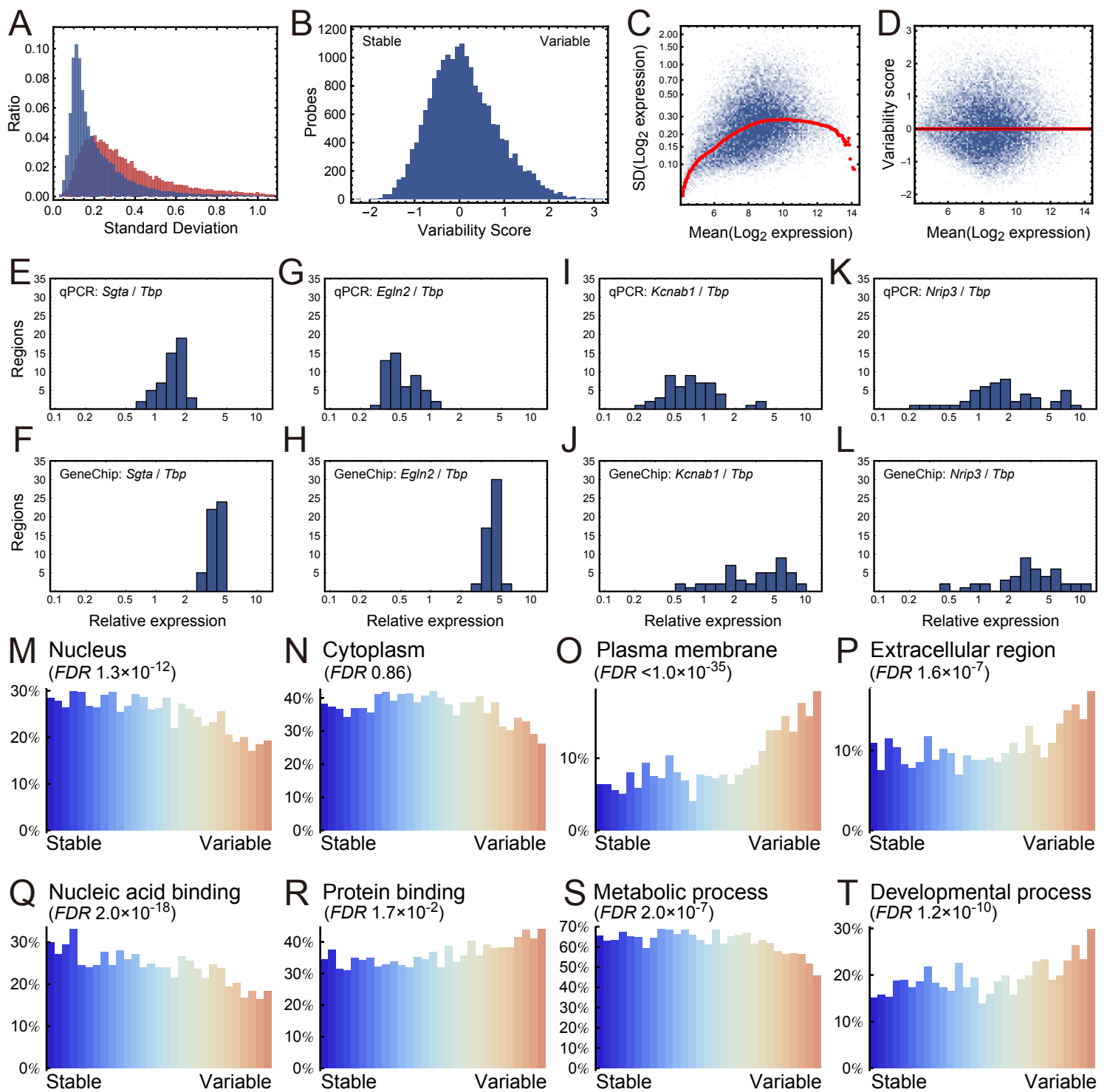

Figure S3 Kasukawa *et al.*

Supplement: Figure S3 — One-state genes. (A) Distribution of the standard deviation of one-state (blue) and multi-state (red) genes. (B) Distribution of variability scores. The higher and lower variability scores of genes indicated that their expression levels were variable and stable, respectively. (C) Scatter plot of the standard deviations against the means of the log2-transformed expression values. Blue dots represent single probe sets, and the red curve shows their running median. (D) Scatter plot of the mean of the log2-transformed expression values and the variability scores. (E–L) Confirmation of several stable and variable one-state genes by q-PCR. The expression values relative to the Tbp expression are shown. Stable one-state genes Sgta (F) and Egln2 (H) were also stable by q-PCR (E and G, respectively), whereas the variable one-state genes Kcnab1 (J) and Nrip3 (L) were also variable by q-PCR (I and K, respectively). (M–T) Correlation of the variability score with the subcellular localization (M–P), molecular function (Q, R), and biological process (S, T) of the gene products. Each graph represents the ratios of genes associated with the Gene Ontology term for 25 subsets of one-state genes, sorted by the rank of their variability scores. The false discovery rate (FDR) for the enrichment of gene functions in stable or variable genes are also shown. (PDF) [file pone.0023228.s003.pdf]

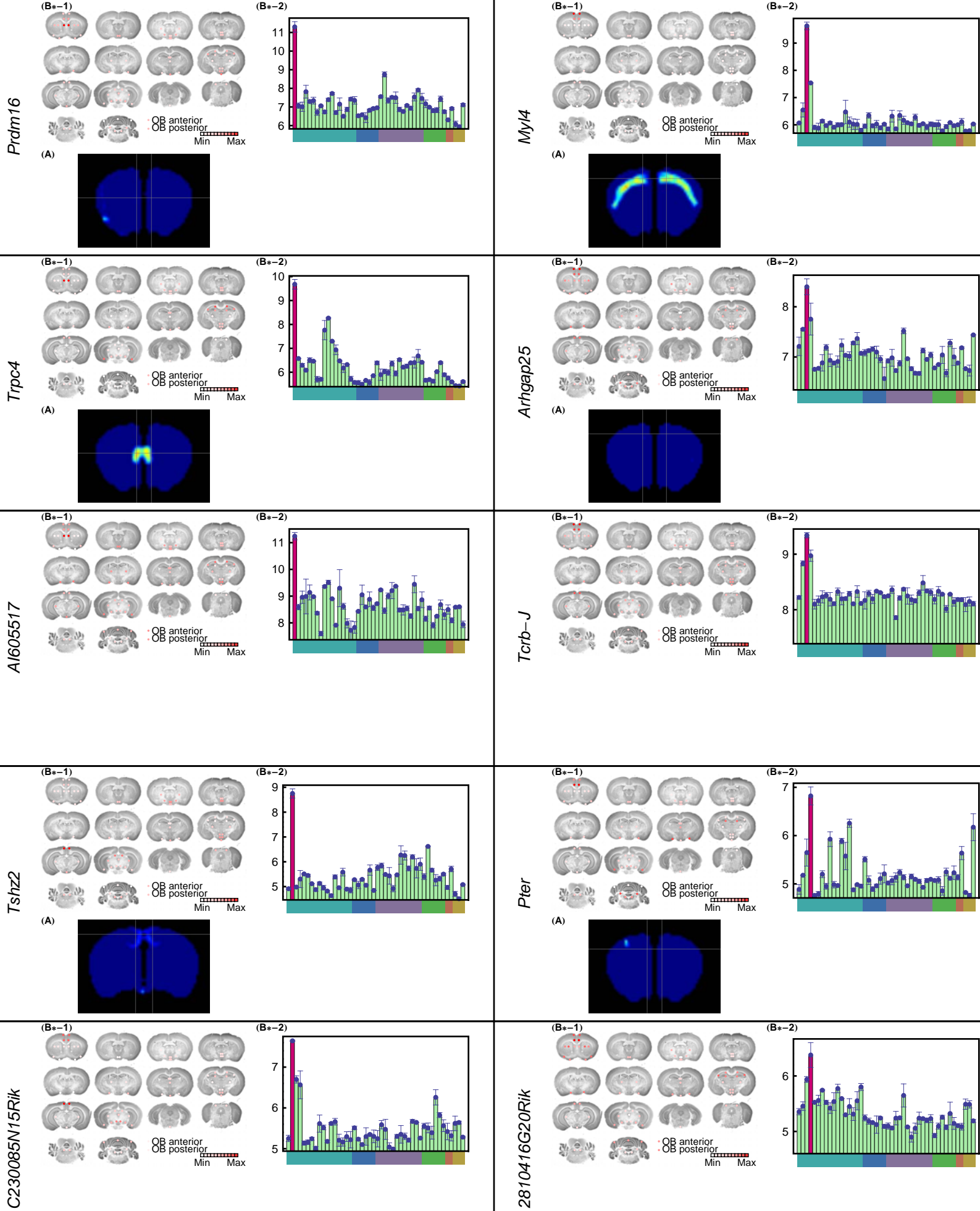

Figure S4 (1/12) Kasukawa *et al.*

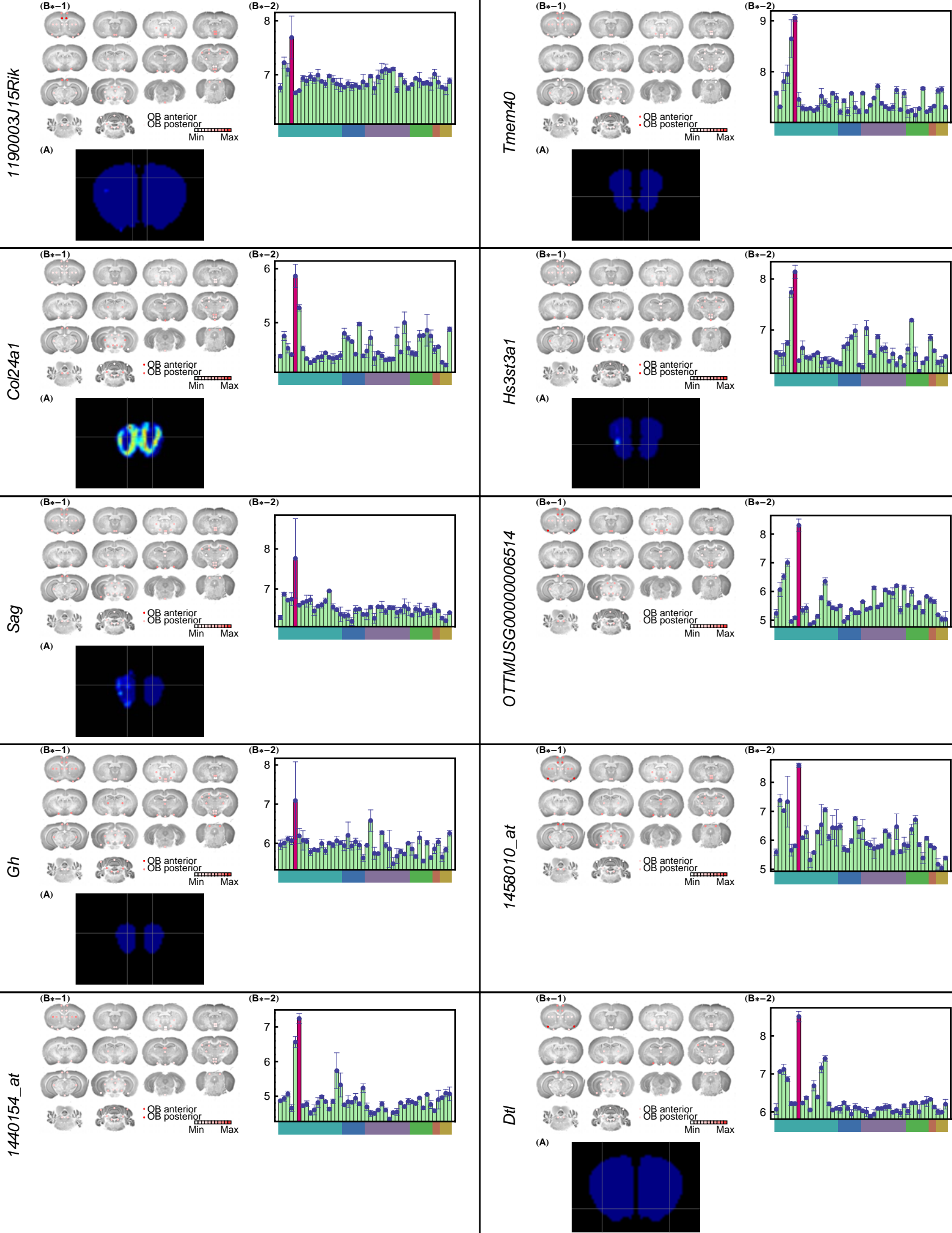

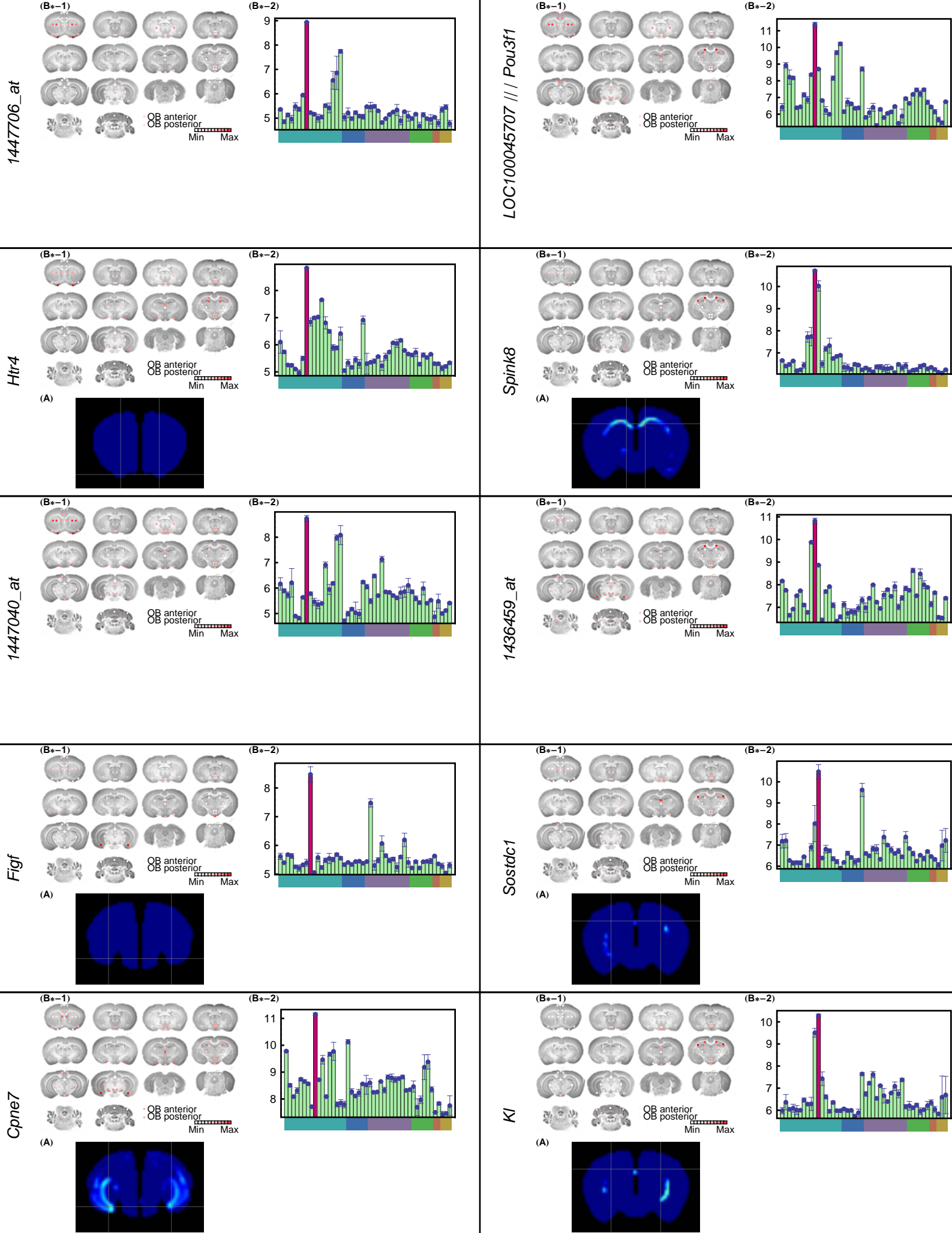

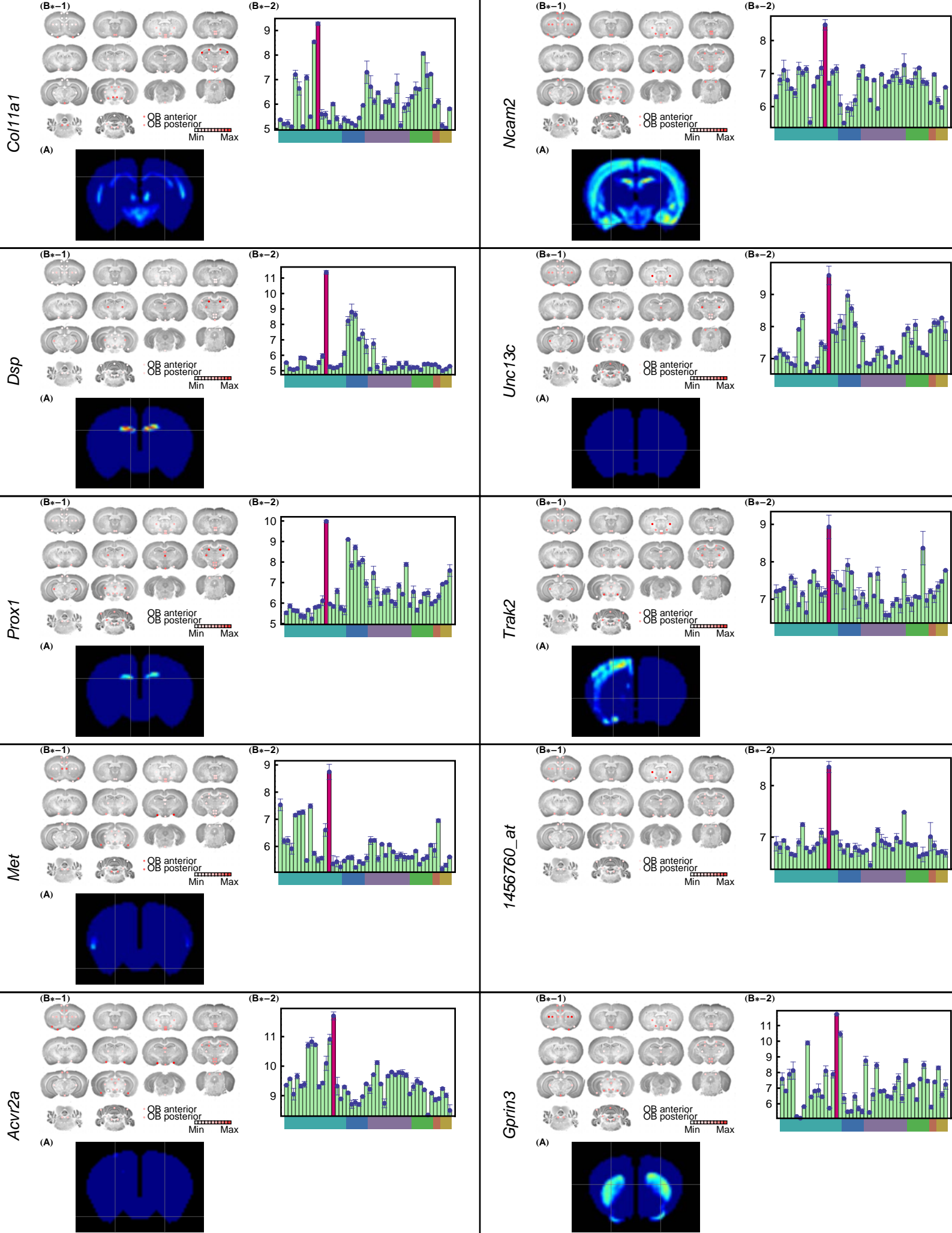

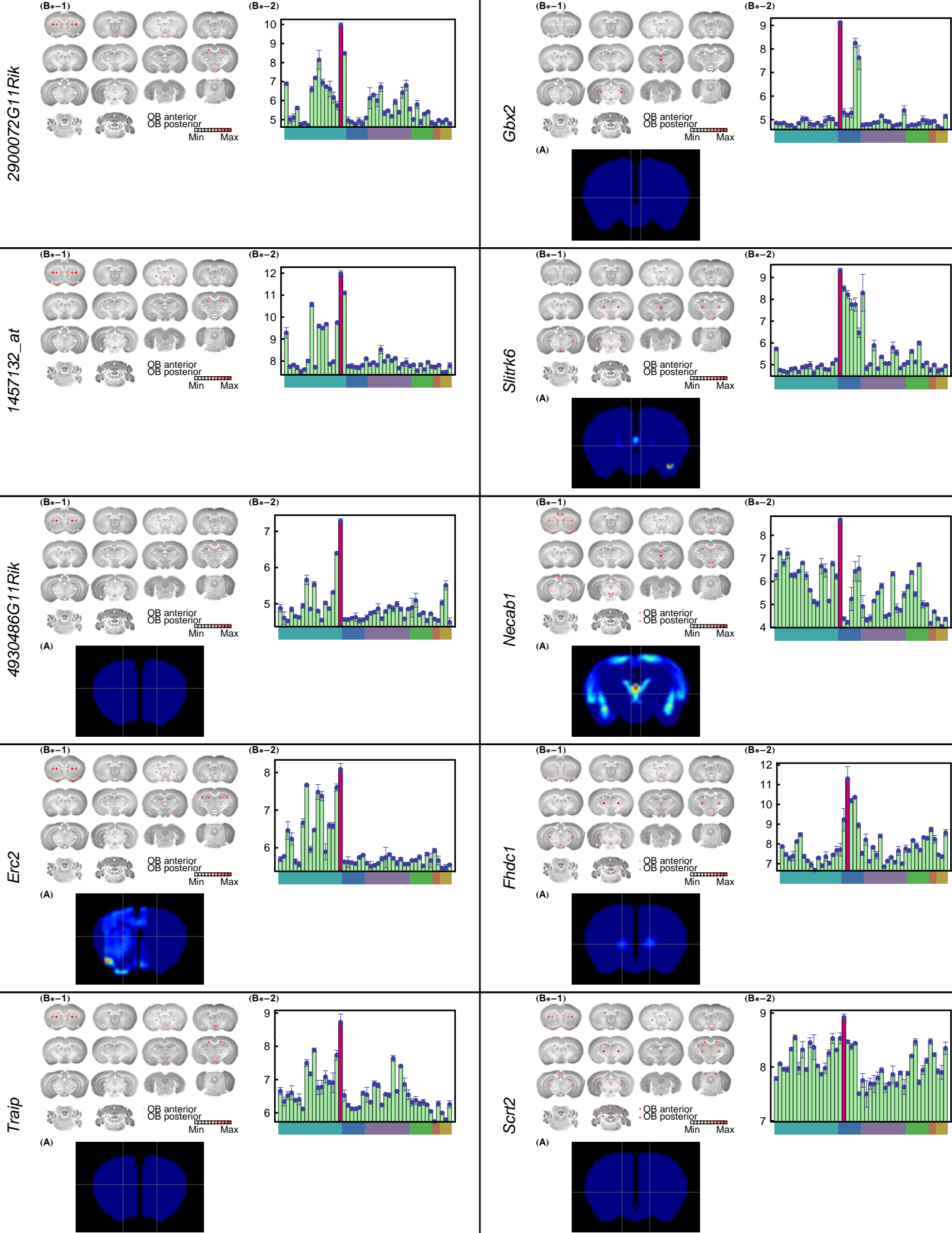

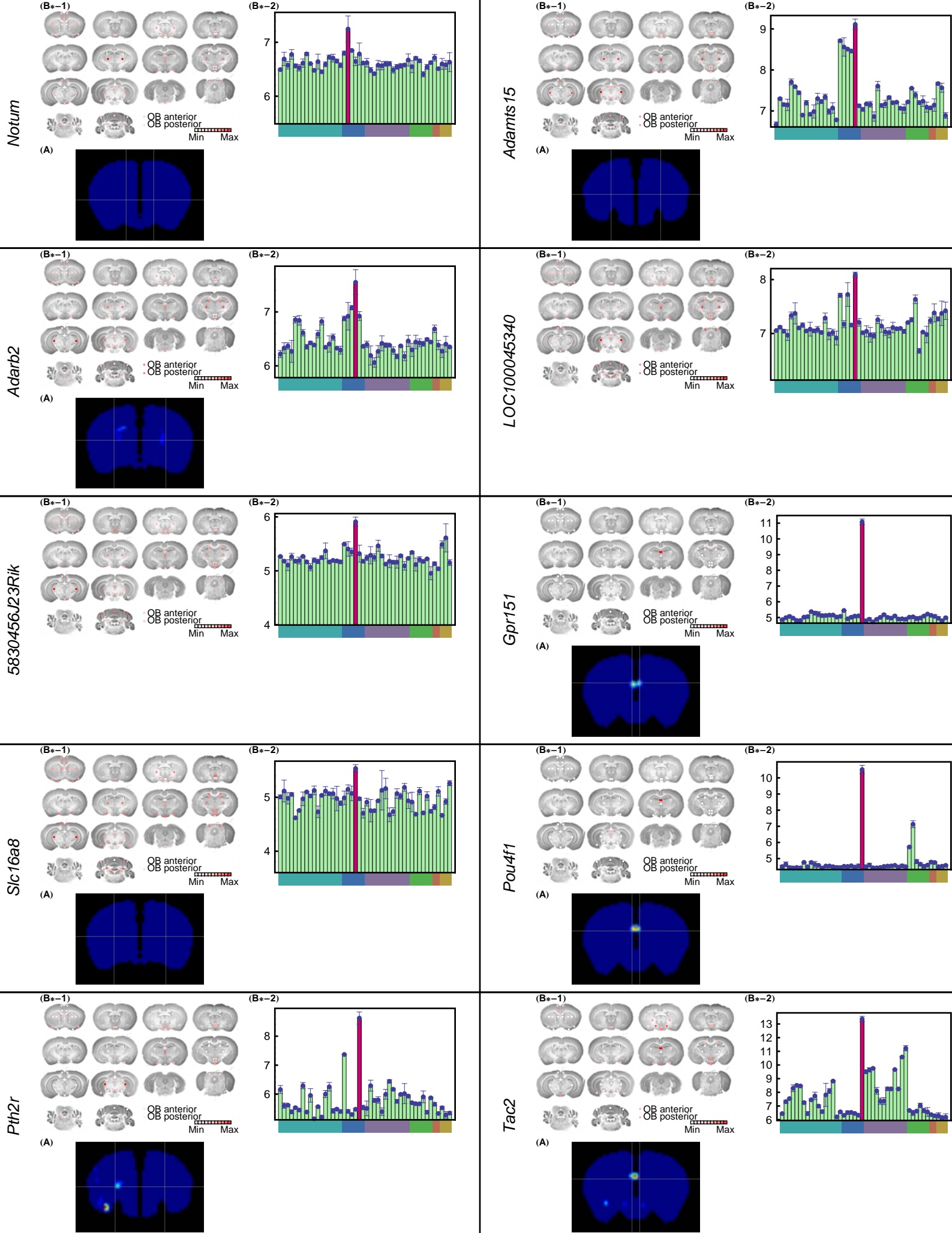

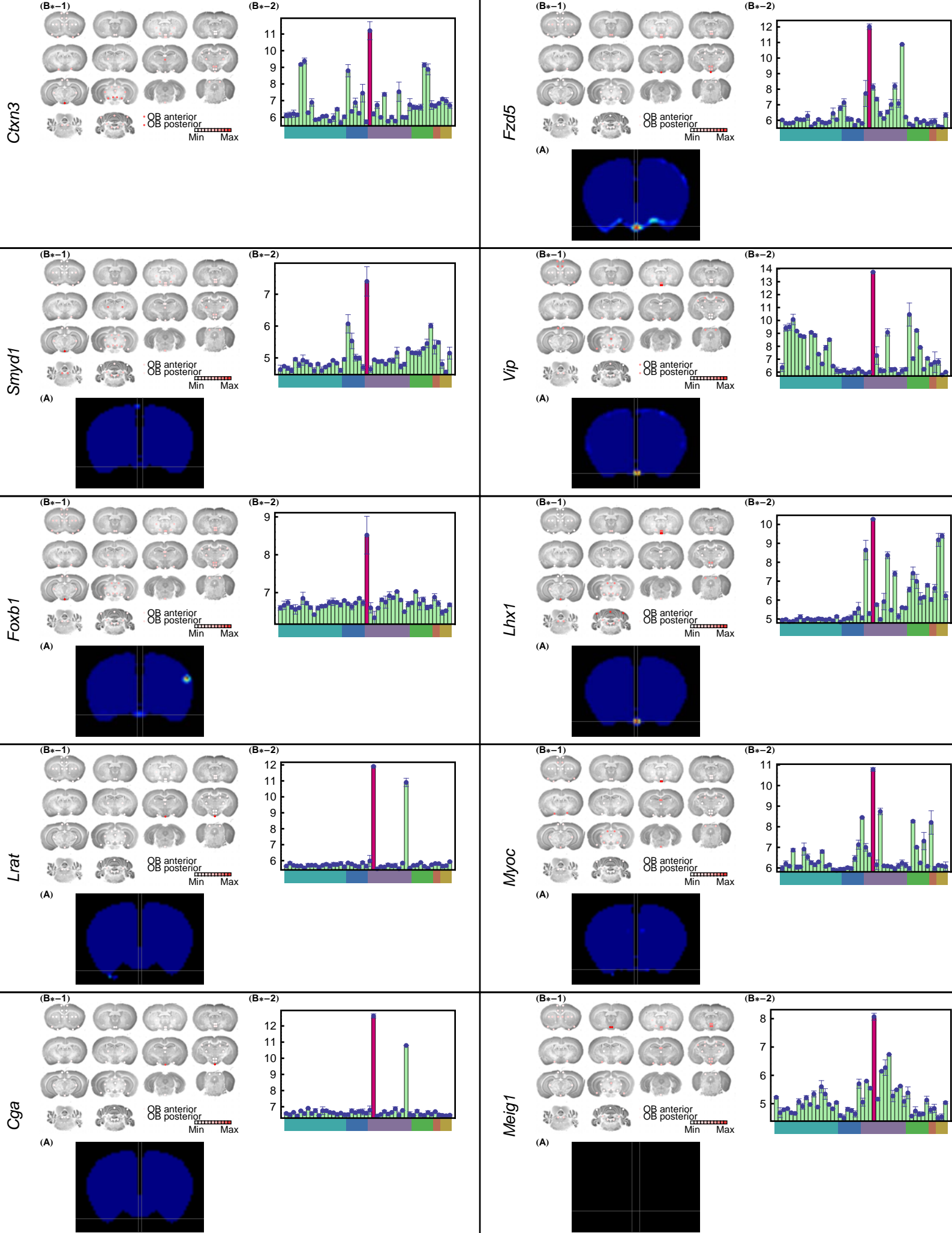

Figure S4 (7/12) Kasukawa *et al.*

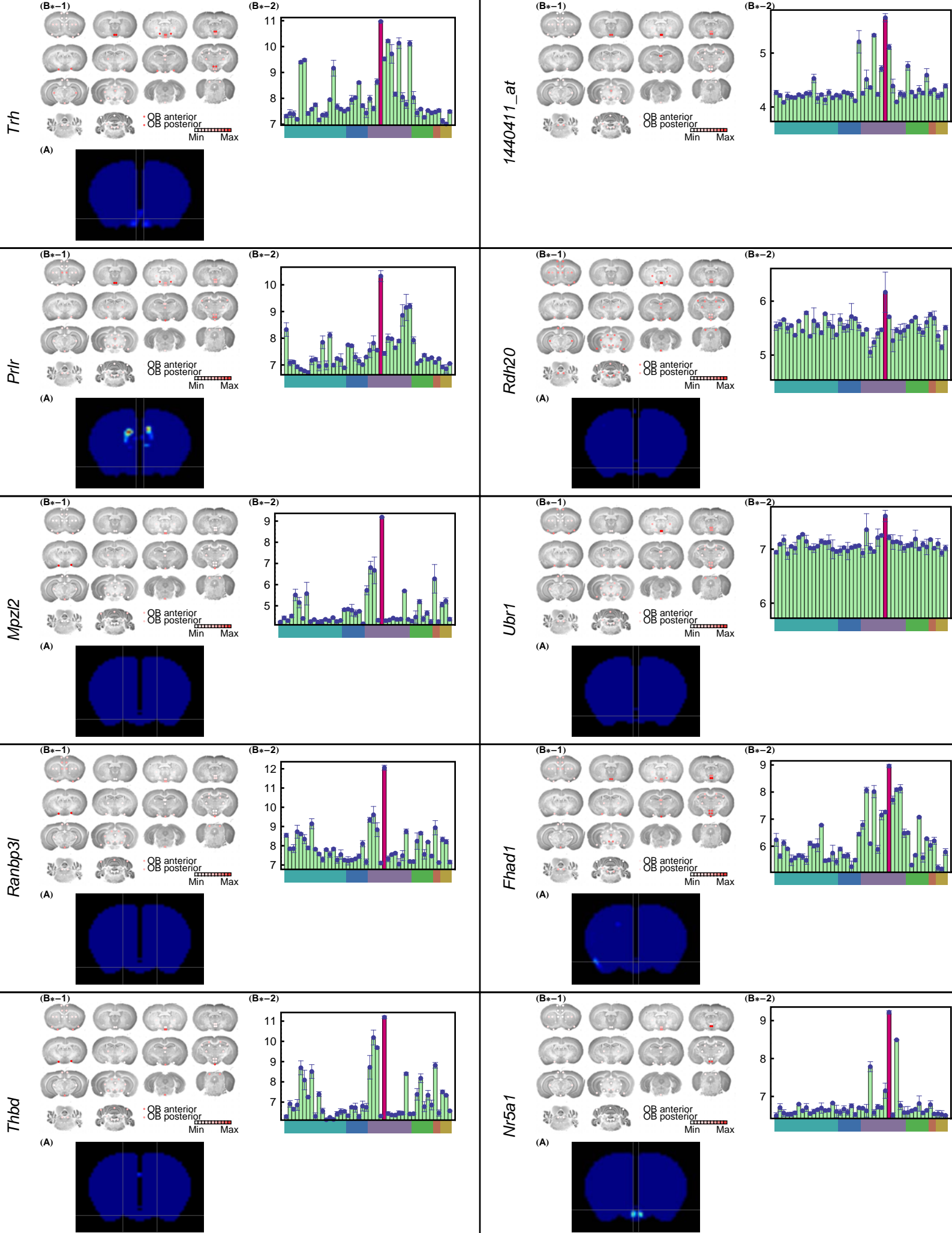

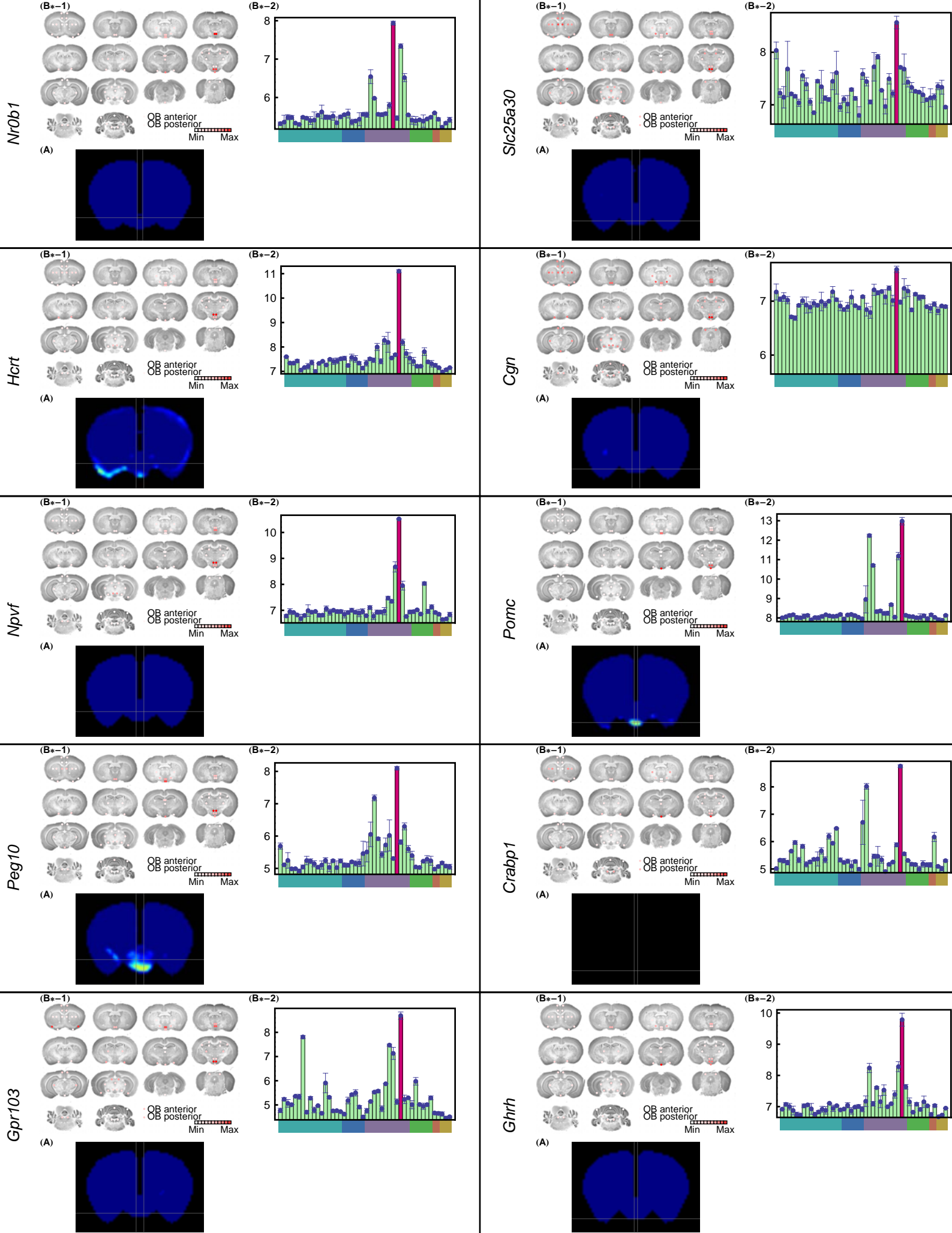

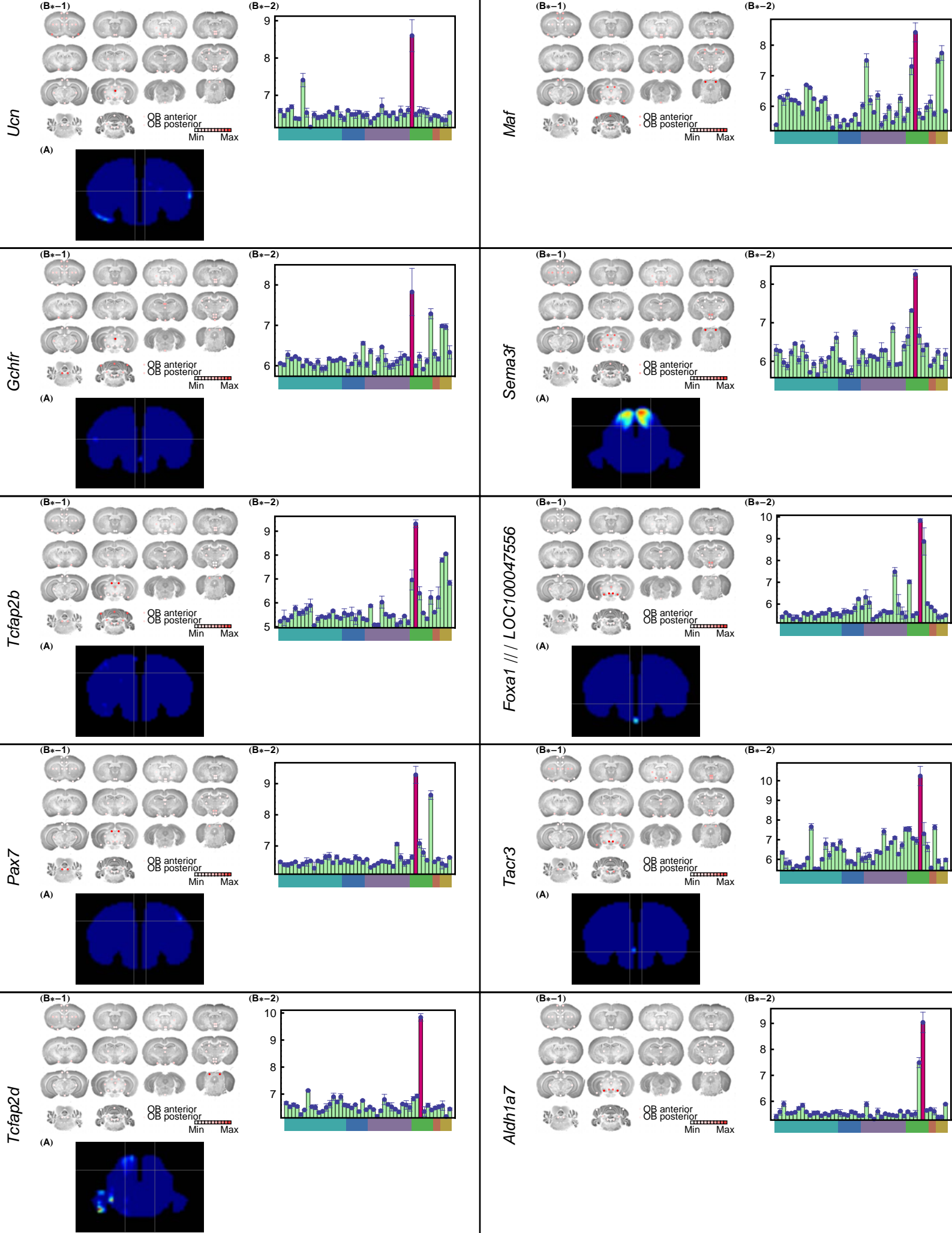

Figure S4 (10/12) Kasukawa *et al.*

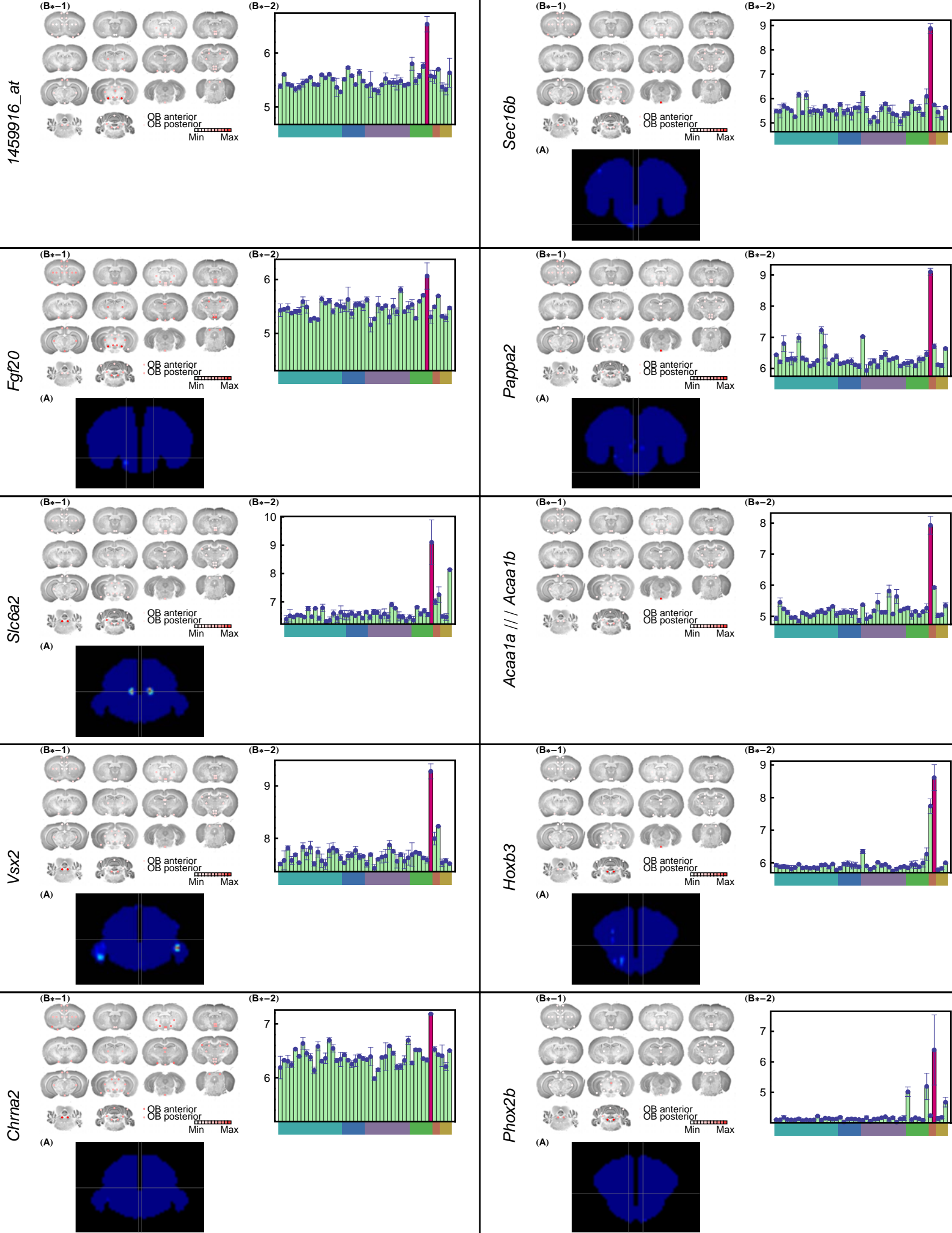

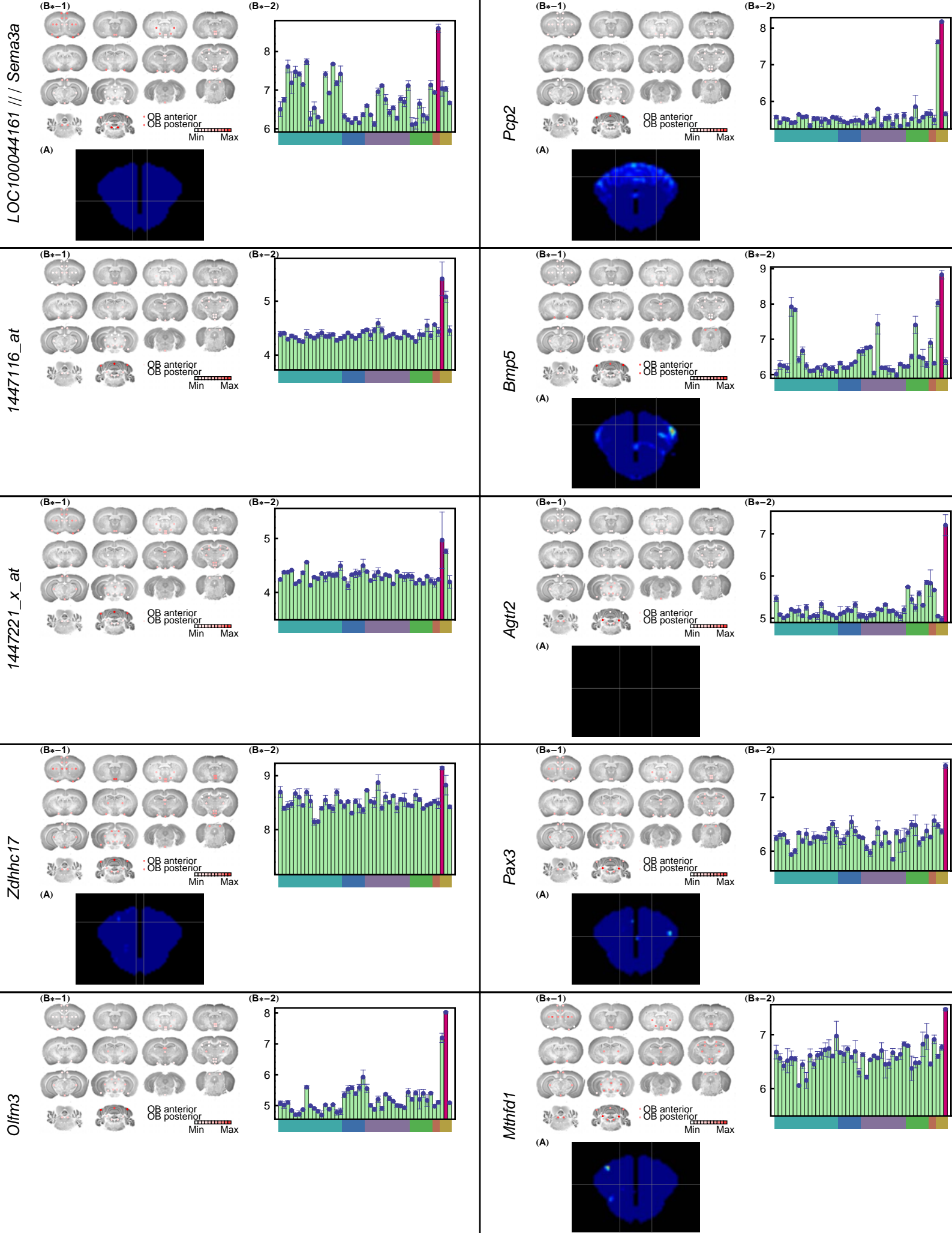

Figure S4 (12/12) Kasukawa *et al.*

Supplement: Figure S4 — Comparison of BrainStars dataset with other resources. Candidates for 120 marker genes from the BrainStars dataset are shown along with results from the Allen Brain Atlas (ABA) dataset. For each gene, the BrainStars expression values were mapped onto images of brain slices (upper-left) and represented in a bar chart (upper-right), and the ABA expression values (“expression energies”) from a coronal cross-section at the expressing CNS region are shown (lower-left). (PDF) [file pone.0023228.s004.pdf]

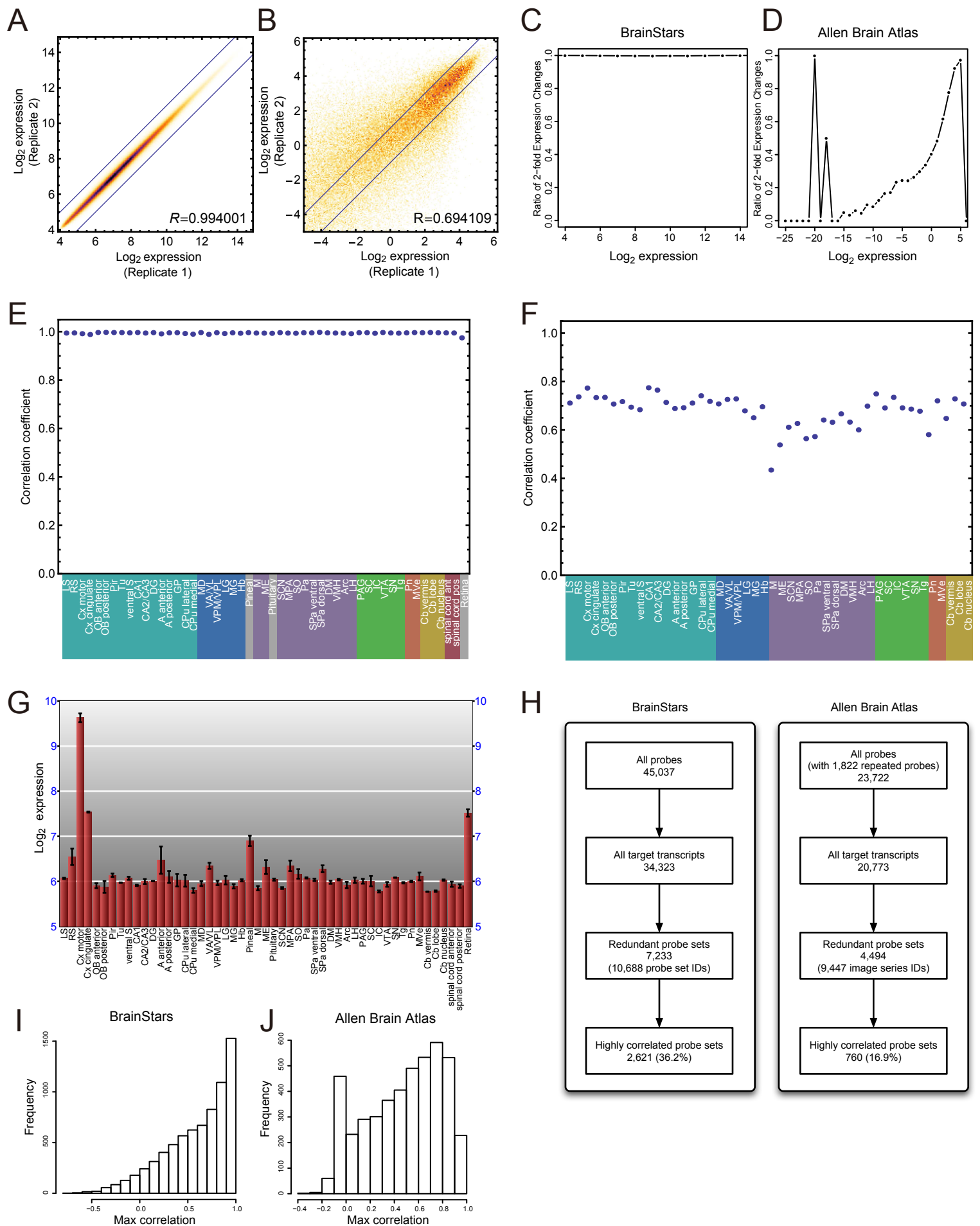

Figure S5 Kasukawa *et al.*

Supplement: Figure S5 — Comparison of reproducibility and quantitativeness between BrainStars and ABA datasets. (A–B) Scatter plot showing the reproducibility of the experimental replicates of all the CNS regions in the BrainStars (A) and Allen Brain Atlas (B) projects. Blue lines indicate 2-fold changes. (C–D) The proportion of replicated data points showing a difference within 2-fold in the BrainStars (C) and Allen Brain Atlas (D) projects. The dynamic range, which we defined as the range of more than 50% of replicated data points that showed a less-than 2-fold change, was from 24 to 214 (∼103.0-fold) for the BrainStars project and 22 to 25 (∼100.9-fold) for the ABA project. (E–F) Correlation coefficient showing the reproducibility of experimental replicates of each CNS region in the BrainStars (E) and Allen Brain Atlas (F) projects. (G) Myl4 expression at 51 CNS regions in our data. Error bars show standard errors. In the Cx motor and Cx cingulate, the Myl4 expressions were greatly changed with small standard errors. (H) Number of redundant probes for the same transcript in each database. (I–J) Maximum correlation coefficient between redundant probes for the same transcript. (I) Distribution of the maximum correlation coefficients for the BrainStars oligo-probes. (J) Distribution of the maximum correlation coefficients in the Allen Brain Atlas. (PDF) [file pone.0023228.s005.pdf]
