# Supplementary material for: Quantitative Expression Profile of Distinct Functional Regions in the Adult Mouse Brain
Source: PLoS One. 2011 Aug 12;6(8):e23228. doi: 10.1371/journal.pone.0023228 (PMC3155528; doi:10.1371/journal.pone.0023228)
Supplement: Table S1 — Sample information for all 51 central nervous system (CNS) regions. (DOC) [file pone.0023228.s006.doc]

Supplementary Table S1. Sample information for all 51 central nervous system (CNS) regions.

(A) CNS regions

|  | Region ID | Region symbol | Region name | Region abbreviation | Region Classification | Note |
| --- | --- | --- | --- | --- | --- | --- |
| 1 | P1.05 | LS | lateral septal nucleus | LS | telencephalon | projection; reinforcement |
| 2 | P2.15 | RS | retrosplenial cortex | RS | telencephalon | projection |
| 3 | P1.01 | Cx_motor | cerebral cortex motor | Cx motor | telencephalon | motion |
| 4 | P1.03 | Cx_cingulate | cerebral cortex cingulate | Cx cingulate | telencephalon | emotion |
| 5 | P3.05 | OB_anterior | olfactory bulb anterior | OB anterior | telencephalon | olfaction |
| 6 | P3.06 | OB_posterior | olfactory bulb posterior | OB posterior | telencephalon | olfaction |
| 7 | P4.01 | Pir | piriform cortex | Pir | telencephalon | olfaction |
| 8 | P2.10 | Tu | olfactory tubercle | Tu | telencephalon | olfaction |
| 9 | P3.15 | ventral_S | ventral subiculum | ventral S | telencephalon | projection from hippocampus |
| 10 | P3.01 | CA1 | CA1 (hippocampus) | CA1 | telencephalon | memory; emotion |
| 11 | P3.08 | CA2_CA3 | CA2 / CA3 (hippocampus) | CA2/CA3 | telencephalon | memory; emotion |
| 12 | P2.04 | DG | dentate gyrus (hippocampus) | DG | telencephalon | memory; emotion; neurogenesis |
| 13 | P1.18 | A_anterior | amygdala anterior | A anterior | telencephalon | emotion; fear; anxiety |
| 14 | P2.12 | A_posterior | amygdala posterior | A posterior | telencephalon | emotion; fear; anxiety |
| 15 | P2.05 | GP | globus pallidus | GP | telencephalon | motion; a part of basal ganglia |
| 16 | P1.06 | CPu_lateral | caudate putamen lateral | CPu lateral | telencephalon | motion; learning |
| 17 | P1.07 | CPu_medial | caudate putamen medial | CPu medial | telencephalon | motion; learning |
| 18 | P2.07 | MD | mediodorsal thalamic nucleus | MD | thalamus | emotion; sensory |
| 19 | P1.17 | VA_VL | ventral anterior thalamic nucleus / ventrolateral thalamic nucleus | VA/VL | thalamus | sensory |
| 20 | P2.18 | VPM_VPL | ventral posteromedial thalamic nucleus / ventral posterolateral thalamic nucleus | VPM/VPL | thalamus | sensory |
| 21 | P1.A | LG | lateral geniculate body | LG | thalamus | vision |
| 22 | P2.09 | MG | medial geniculate nucleus | MG | thalamus | audition |
| 23 | P3.09 | Hb | habenular nucleus | Hb | thalamus | behaviour |
| 24 | P5.03 | Pineal | corpus pineal | Pineal | misc | wake/sleep; melatonin |
| 25 | P1.22 | M | mammillary body | M | hypothalamus | emotion; Papez's circuit |
| 26 | P4.02 | ME | median eminence | ME | hypothalamus | endocrine; photoperiodic calendar |
| 27 | P3.07 | Pituitary | pituitary | Pituitary | misc | endocrine |
| 28 | P3.03 | SCN | suprachiasmatic nucleus | SCN | hypothalamus | circadian clock center |
| 29 | P1.12 | MPA | medial preoptic area | MPA | hypothalamus | thermoregulation |
| 30 | P1.14 | SO | supraoptic nucleus | SO | hypothalamus | vasopressin; oxytocin |
| 31 | P2.13 | Pa | paraventricular hypothalamic nucleus | Pa | hypothalamus | stress; vasopressin; oxytocin; CRF |
| 32 | P4.03 | SPa_ventral | subparaventricular zone ventral | SPa ventral | hypothalamus | projection from SCN |
| 33 | P5.01 | SPa_dorsal | subparaventricular zone dorsal | SPa dorsal | hypothalamus | projection from SCN |
| 34 | P3.02 | DM | dorsomedial hypothalamic nucleus | DM | hypothalamus | feeding; behaviour |
| 35 | P1.20 | VMH | ventromedial hypothalamic nucleus | VMH | hypothalamus | satiety center |
| 36 | P2.01 | Arc | arcuate hypothalamic nucleus | Arc | hypothalamus | endocrine; projection |
| 37 | P1.13 | LH | lateral hypothalamus | LH | hypothalamus | hunger center |
| 38 | P2.11 | PAG | periaqueductal gray | PAG | mesencephalon | pain regulation |
| 39 | P3.12 | SC | superior colliculus | SC | mesencephalon | vision; sensory |
| 40 | P2.06 | IC | inferior colliculus | IC | mesencephalon | audition |
| 41 | P5.02 | VTA | ventral tegmental area | VTA | mesencephalon | reward; dopamine |
| 42 | P1.28 | SN | substantia nigra | SN | mesencephalon | reward; dopamine |
| 43 | P3.14 | Tg | dorsal tegmental nucleus | Tg | mesencephalon | acetylcholine; REM sleep |
| 44 | P3.13 | Pn | pontine nucleus | Pn | pons | motion |
| 45 | P2.08 | MVe | medial vestibular nucleus | MVe | pons | equilibrium |
| 46 | P3.10 | Cb_vermis | cerebellar cortex vermis | Cb vermis | cerebellum | motion |
| 47 | P3.11 | Cb_lobe | cerebellar cortex lobe | Cb lobe | cerebellum | motion |
| 48 | P2.03 | Cb_nucleus | cerebellar nucleus | Cb nucleus | cerebellum | output from cerebellum |
| 49 | P2.16 | spinal_cord_anterior | spinal cord anterior | spinal cord anterior | spinal cord | motor neurons |
| 50 | P2.17 | spinal_cord_posterior | spinal cord posterior | spinal cord posterior | spinal cord | touch |
| 51 | P3.04 | Retina | retina | Retina | misc | vision |

The information for CNS regions provides the region ID, region symbol, region name, region abbreviation, anatomical classification, and note for each region.

(B) sample information

| Sample ID | Region ID | Region symbol | Region abbreviation | #mice | Sampling date1 | Sampling date2 |
| --- | --- | --- | --- | --- | --- | --- |
| P1.05.1 | P1.05 | LS | LS | 5 | 2005/4/27 |  |
| P1.05.2 | P1.05 | LS | LS | 5 | 2005/5/1 | 2005/5/14 |
| P2.15.1 | P2.15 | RS | RS | 5 | 2005/9/27 |  |
| P2.15.2 | P2.15 | RS | RS | 5 | 2005/10/4 |  |
| P1.01.1 | P1.01 | Cx_motor | Cx motor | 5 | 2005/4/27 |  |
| P1.01.2 | P1.01 | Cx_motor | Cx motor | 5 | 2005/5/1 |  |
| P1.03.1 | P1.03 | Cx_cingulate | Cx cingulate | 5 | 2005/4/27 |  |
| P1.03.2 | P1.03 | Cx_cingulate | Cx cingulate | 5 | 2005/5/1 |  |
| P3.05.1 | P3.05 | OB_anterior | OB anterior | 5 | 2006/7/25 |  |
| P3.05.2 | P3.05 | OB_anterior | OB anterior | 5 | 2006/7/27 |  |
| P3.06.1 | P3.06 | OB_posterior | OB posterior | 5 | 2006/7/25 |  |
| P3.06.2 | P3.06 | OB_posterior | OB posterior | 5 | 2006/7/27 |  |
| P4.01.1 | P4.01 | Pir | Pir | 15 | 2006/12/14 |  |
| P4.01.2 | P4.01 | Pir | Pir | 15 | 2006/12/14 |  |
| P2.10.1 | P2.10 | Tu | Tu | 15 | 2005/7/29 |  |
| P2.10.2 | P2.10 | Tu | Tu | 15 | 2005/8/15 |  |
| P3.15.1 | P3.15 | ventral_S | ventral S | 15 | 2006/7/25 |  |
| P3.15.2 | P3.15 | ventral_S | ventral S | 15 | 2006/7/27 |  |
| P3.01.1 | P3.01 | CA1 | CA1 | 15 | 2006/6/23 |  |
| P3.01.2 | P3.01 | CA1 | CA1 | 15 | 2006/7/26 |  |
| P3.08.1 | P3.08 | CA2_CA3 | CA2/CA3 | 15 | 2006/6/23 |  |
| P3.08.2 | P3.08 | CA2_CA3 | CA2/CA3 | 15 | 2006/7/27 |  |
| P2.04.1 | P2.04 | DG | DG | 15 | 2005/8/23 |  |
| P2.04.2 | P2.04 | DG | DG | 15 | 2005/8/30 |  |
| P1.18.1 | P1.18 | A_anterior | A anterior | 15 | 2005/4/27 |  |
| P1.18.2 | P1.18 | A_anterior | A anterior | 15 | 2005/5/1 |  |
| P2.12.1 | P2.12 | A_posterior | A posterior | 15 | 2005/5/29 | 2005/5/31 |
| P2.12.2 | P2.12 | A_posterior | A posterior | 15 | 2005/6/8 |  |
| P2.05.1 | P2.05 | GP | GP | 15 | 2005/8/23 |  |
| P2.05.2 | P2.05 | GP | GP | 15 | 2005/8/30 |  |
| P1.06.1 | P1.06 | CPu_lateral | CPu lateral | 5 | 2005/4/27 |  |
| P1.06.2 | P1.06 | CPu_lateral | CPu lateral | 5 | 2005/5/1 | 2005/5/14 |
| P1.07.1 | P1.07 | CPu_medial | CPu medial | 5 | 2005/4/27 |  |
| P1.07.2 | P1.07 | CPu_medial | CPu medial | 5 | 2005/5/1 | 2005/5/14 |
| P2.07.1 | P2.07 | MD | MD | 15 | 2005/8/23 |  |
| P2.07.2 | P2.07 | MD | MD | 15 | 2005/8/30 |  |
| P1.17.1 | P1.17 | VA_VL | VA/VL | 15 | 2005/4/27 | 2005/5/1 |
| P1.17.2 | P1.17 | VA_VL | VA/VL | 15 | 2005/5/14 | 2005/6/8 |
| P2.18.1 | P2.18 | VPM_VPL | VPM/VPL | 15 | 2005/8/23 |  |
| P2.18.2 | P2.18 | VPM_VPL | VPM/VPL | 15 | 2005/8/30 |  |
| P1.A.1 | P1.A | LG | LG | 15 | 2005/5/1 | 2005/5/14 |
| P1.A.2 | P1.A | LG | LG | 15 | 2005/5/31 | 2005/6/8 |
| P2.09.1 | P2.09 | MG | MG | 15 | 2005/9/30 |  |
| P2.09.2 | P2.09 | MG | MG | 15 | 2005/10/4 |  |
| P3.09.1 | P3.09 | Hb | Hb | 15 | 2006/6/23 |  |
| P3.09.2 | P3.09 | Hb | Hb | 15 | 2006/7/26 |  |
| P5.03.1 | P5.03 | Pineal | Pineal | 5 | 2007/2/20 |  |
| P5.03.2 | P5.03 | Pineal | Pineal | 5 | 2007/2/20 |  |
| P1.22.1 | P1.22 | M | M | 15 | 2005/5/1 | 2005/5/14 |
| P1.22.2 | P1.22 | M | M | 15 | 2005/5/29 | 2005/5/31 |
| P4.02.1 | P4.02 | ME | ME | 25 | 2006/12/14 |  |
| P4.02.2 | P4.02 | ME | ME | 25 | 2006/12/14 |  |
| P3.07.1 | P3.07 | Pituitary | Pituitary | 5 | 2006/5/24 |  |
| P3.07.2 | P3.07 | Pituitary | Pituitary | 5 | 2006/7/27 |  |
| P3.03.1 | P3.03 | SCN | SCN | 15 | 2006/7/26 | 2006/7/27 |
| P3.03.2 | P3.03 | SCN | SCN | 15 | 2006/8/10 |  |
| P1.12.1 | P1.12 | MPA | MPA | 15 | 2005/4/27 | 2005/5/1 |
| P1.12.2 | P1.12 | MPA | MPA | 15 | 2005/5/14 | 2005/5/29 |
| P1.14.1 | P1.14 | SO | SO | 15 | 2005/4/27 | 2005/5/1 |
| P1.14.2 | P1.14 | SO | SO | 15 | 2005/5/14 | 2005/5/31 |
| P2.13.1 | P2.13 | Pa | Pa | 15 | 2005/7/29 | 2005/8/23 |
| P2.13.2 | P2.13 | Pa | Pa | 15 | 2005/8/15 | 2005/8/30 |
| P4.03.1 | P4.03 | SPa_ventral | SPa ventral | 15 | 2007/2/20 |  |
| P4.03.2 | P4.03 | SPa_ventral | SPa ventral | 15 | 2007/2/20 |  |
| P5.01.1 | P5.01 | SPa_dorsal | SPa dorsal | 15 | 2007/2/20 |  |
| P5.01.2 | P5.01 | SPa_dorsal | SPa dorsal | 15 | 2007/2/20 |  |
| P3.02.1 | P3.02 | DM | DM | 15 | 2006/7/26 |  |
| P3.02.2 | P3.02 | DM | DM | 15 | 2006/8/10 |  |
| P1.20.1 | P1.20 | VMH | VMH | 15 | 2005/4/27 | 2005/5/1 |
| P1.20.2 | P1.20 | VMH | VMH | 15 | 2005/5/29 | 2005/5/31 |
| P2.01.1 | P2.01 | Arc | Arc | 15 | 2005/9/22 | 2005/9/30 |
| P2.01.2 | P2.01 | Arc | Arc | 15 | 2005/9/27 | 2005/10/4 |
| P1.13.1 | P1.13 | LH | LH | 15 | 2005/4/27 | 2005/5/1 |
| P1.13.2 | P1.13 | LH | LH | 15 | 2005/5/14 | 2005/5/29 |
| P2.11.1 | P2.11 | PAG | PAG | 15 | 2005/8/23 |  |
| P2.11.2 | P2.11 | PAG | PAG | 15 | 2005/8/30 |  |
| P3.12.1 | P3.12 | SC | SC | 15 | 2005/9/27 |  |
| P3.12.2 | P3.12 | SC | SC | 15 | 2005/9/30 |  |
| P2.06.1 | P2.06 | IC | IC | 15 | 2005/9/30 |  |
| P2.06.2 | P2.06 | IC | IC | 15 | 2005/10/4 |  |
| P5.02.1 | P5.02 | VTA | VTA | 15 | 2007/2/20 |  |
| P5.02.2 | P5.02 | VTA | VTA | 15 | 2007/2/20 |  |
| P1.28.1 | P1.28 | SN | SN | 15 | 2005/5/1 | 2005/5/29 |
| P1.28.2 | P1.28 | SN | SN | 15 | 2005/5/31 |  |
| P3.14.1 | P3.14 | Tg | Tg | 15 | 2005/9/22 | 2005/9/30 |
| P3.14.2 | P3.14 | Tg | Tg | 15 | 2005/9/30 |  |
| P3.13.1 | P3.13 | Pn | Pn | 15 | 2005/9/27 |  |
| P3.13.2 | P3.13 | Pn | Pn | 15 | 2005/9/30 | 2005/10/4 |
| P2.08.1 | P2.08 | MVe | MVe | 15 | 2005/9/22 | 2005/9/27 |
| P2.08.2 | P2.08 | MVe | MVe | 15 | 2005/9/30 |  |
| P3.10.1 | P3.10 | Cb_vermis | Cb vermis | 5 | 2005/5/1 |  |
| P3.10.2 | P3.10 | Cb_vermis | Cb vermis | 5 | 2005/5/14 |  |
| P3.11.1 | P3.11 | Cb_lobe | Cb lobe | 5 | 2005/5/1 |  |
| P3.11.2 | P3.11 | Cb_lobe | Cb lobe | 5 | 2005/5/14 |  |
| P2.03.1 | P2.03 | Cb_nucleus | Cb nucleus | 15 | 2005/9/27 |  |
| P2.03.2 | P2.03 | Cb_nucleus | Cb nucleus | 15 | 2005/9/30 | 2005/10/4 |
| P2.16.1 | P2.16 | spinal_cord_anterior | spinal cord anterior | 15 | 2005/8/23 |  |
| P2.16.2 | P2.16 | spinal_cord_anterior | spinal cord anterior | 15 | 2005/8/30 |  |
| P2.17.1 | P2.17 | spinal_cord_posterior | spinal cord posterior | 15 | 2005/8/23 |  |
| P2.17.2 | P2.17 | spinal_cord_posterior | spinal cord posterior | 15 | 2005/8/30 |  |
| P3.04.1 | P3.04 | Retina | Retina | 5 | 2006/5/24 |  |
| P3.04.2 | P3.04 | Retina | Retina | 5 | 2006/7/27 |  |

The information for samples includes the sample ID, region ID, region symbol, number of sampled mice for each time point, and sample dates for each experimental replicate.
