# Supplementary material for: Quantitative Expression Profile of Distinct Functional Regions in the Adult Mouse Brain
Source: PLoS One. 2011 Aug 12;6(8):e23228. doi: 10.1371/journal.pone.0023228 (PMC3155528; doi:10.1371/journal.pone.0023228)
Supplement: Table S2 — Pearson's correlation coefficients of the BrainStars dataset with the Allen Brain Atlas (ABA) dataset. (DOC) [file pone.0023228.s007.doc]

Supplementary Table S2. Pearson’s correlation coefficients of the BrainStars dataset with the Allen Brain Atlas (ABA) dataset.

| Region | Correlation with ABA (expression energy) |
| --- | --- |
| All regions | 0.454 |
| LS | 0.497 |
| RS | 0.492 |
| Cx motor | 0.537 |
| Cx cingulate | 0.529 |
| OB anterior | 0.460 |
| OB posterior | 0.449 |
| Pir | 0.495 |
| Tu | 0.471 |
| ventral S | 0.479 |
| CA1 | 0.558 |
| CA2/CA3 | 0.542 |
| DG | 0.506 |
| A anterior | 0.449 |
| A posterior | 0.453 |
| GP | 0.480 |
| CPu lateral | 0.492 |
| CPu medial | 0.498 |
| MD | 0.485 |
| VA/VL | 0.490 |
| VPM/VPL | 0.492 |
| LG | 0.481 |
| MG | 0.432 |
| Hb | 0.504 |
| M | 0.347 |
| ME | 0.369 |
| SCN | 0.406 |
| MPA | 0.430 |
| SO | 0.316 |
| Pa | 0.471 |
| SPa ventral | 0.436 |
| SPa dorsal | 0.451 |
| DM | 0.476 |
| VMH | 0.424 |
| Arc | 0.380 |
| LH | 0.477 |
| PAG | 0.515 |
| SC | 0.471 |
| IC | 0.492 |
| VTA | 0.461 |
| SN | 0.445 |
| Tg | 0.441 |
| Pn | 0.419 |
| MVe | 0.468 |
| Cb vermis | 0.470 |
| Cb lobe | 0.483 |
| Cb nucleus | 0.447 |
