# Supplementary material for: Quantitative Expression Profile of Distinct Functional Regions in the Adult Mouse Brain
Source: PLoS One. 2011 Aug 12;6(8):e23228. doi: 10.1371/journal.pone.0023228 (PMC3155528; doi:10.1371/journal.pone.0023228)
Supplement: Table S3 — Comparison of the marker gene candidates in the BrainStars dataset with the Allen Brain Atlas (ABA) dataset. (DOC) [file pone.0023228.s008.doc]

Supplementary Table S3. Comparison of the marker gene candidates in the BrainStars dataset with the Allen Brain Atlas (ABA) dataset.

| BrainStars probe set ID | highly expressed region | ABA Gene Symbol | ABA ImageSeriesID (coronal) | ABA ImageSeriesID (sagittal) | Gene Symbol | ABA comparison |
| --- | --- | --- | --- | --- | --- | --- |
| 1440870_at | LS | Prdm16 |  | 68845323 | Prdm16 | unconfirmable |
| 1423579_a_at | LS | Trpc4 | 1305 | 79904698 1306 | Trpc4 | matched |
| 1459250_at 1435385_at | RS | Tshz2 | 72129294 | 69133824 | Tshz2 | matched |
| 1422580_at | Cx motor | Myl4 | 72129251 | 79760484 | Myl4 | mismatched |
| 1437072_at | Cx motor | Arhgap25 | 72128789 | 69526815 | Arhgap25 | mismatched |
| 1453578_at | Cx cingulate | Pter |  | 71763849 | Pter | unconfirmable |
| 1455186_a_at | Cx cingulate | 1190003J15Rik |  | 68744038 | 1190003J15Rik | unconfirmable |
| 1453418_at | OB anterior | Col24a1 | 73636093 | 76081093 | Col24a1 | mismatched |
| 1419025_at | OB anterior | Sag |  | 68443375 | Sag | unconfirmable |
| 1460613_x_at | OB anterior | Gh |  | 74513935 | Gh | unconfirmable |
| 1424966_at | OB posterior | Tmem40 |  | 76098295 | Tmem40 | unconfirmable |
| 1435622_at | OB posterior | Hs3st3a1 |  | 69735102 | Hs3st3a1 | unconfirmable |
| 1434695_at | Pir | Dtl |  | 69816428 | Dtl | unconfirmable |
| 1443365_at | Tu | Htr4 | 72119658 | 69257849 | Htr4 | matched |
| 1438954_x_at 1438953_at | ventral S | Figf |  | 68911007 | Figf | unconfirmable |
| 1433715_at | ventral S | Cpne7 | 73817432 | 69608010 | Cpne7 | mismatched |
| 1435999_at | CA1 | C630041L24Rik | 72129253 | 69114085 | Spink8 | matched |
| 1449340_at | CA2/CA3 | Sostdc1 | 77874602 71836808 | 80472065 | Sostdc1 | matched |
| 1423400_at | CA2/CA3 | Kl | 72119590 | 69201576 | Kl | unconfirmable |
| 1449154_at | CA2/CA3 | Col11a1 | 71064195 | 68797828 | Col11a1 | matched |
| 1435494_s_at 1435493_at | DG | Dsp | 76097696 | 69201504 | Dsp | matched |
| 1437894_at | DG | Prox1 | 73520980 | 69289763 | Prox1 | mismatched |
| 1434447_at | A posterior | Met | 79591401 73787949 | 69289351 | Met | mismatched |
| 1437382_at | A posterior | Acvr2a |  | 74509464 | Acvr2a | mismatched |
| 1425301_at | A posterior | Ncam2 | 682 | 683 | Ncam2 | unconfirmable |
| 1430511_at | GP | Unc13c | 2707 | 2708 | Unc13c | unconfirmable |
| 1429607_at | GP | Trak2 |  | 75990672 | Trak2 | unconfirmable |
| 1439566_at | CPu lateral | Gprin3 | 73520968 | 69530756 | Gprin3 | matched |
| 1437935_at | CPu medial | 4930486G11Rik |  | 70717359 | 4930486G11Rik | unconfirmable |
| 1444681_at | CPu medial | D14Ertd171e |  | 68797661 | Erc2 | mismatched |
| 1420707_a_at | CPu medial | Traip | 77887842 | 76097714 | Traip | matched |
| 1420337_at | MD | Gbx2 | 77868786 488 | 490 77464958 489 | Gbx2 | mismatched |
| 1437231_at | MD | Slitrk6 | 73992918 | 69874162 | Slitrk6 | mismatched |
| 1437156_at | MD | Efcbp1 | 72472791 | 69671899 | Necab1 | matched |
| 1439018_at | VA/VL | 6330505N24Rik | 73925717 | 69444861 | Fhdc1 | matched |
| 1440930_a_at | VA/VL | Scrt2 |  | 71211383 | Scrt2 | unconfirmable |
| 1451857_a_at | VA/VL | 5730593N15Rik | 73732151 | 69549168 | Notum | mismatched |
| 1421300_at | LG | Adarb2 | 73925721 | 70946222 | Adarb2 | matched |
| 1457014_x_at | LG | Slc16a8 |  | 74431453 | Slc16a8 | unconfirmable |
| 1452129_at | MG | Pthr2 |  | 69863250 | Pth2r | unconfirmable |
| 1427056_at | MG | Adamts15 | 77332098 | 69514792 | Adamts15 | matched |
| 1457555_at | Hb | Gpr151 | 74724649 | 69257468 | Gpr151 | mismatched |
| 1429668_at | Hb | Pou4f1 | 74641304 | 71280601 | Pou4f1 | mismatched |
| 1419411_at | Hb | Tac2 | 77279001 72339556 | 75694341 | Tac2 | mismatched |
| 1441667_s_at | M | Smyd1 |  | 71488780 | Smyd1 | unconfirmable |
| 1420705_at | M | Foxb1 | 79908799 460 | 461 | Foxb1 | matched |
| 1444487_at | ME | Lrat |  | 70300204 | Lrat | unconfirmable |
| 1418549_at | ME | Cga |  | 69548876 | Cga | unconfirmable |
| 1455604_at | ME | Fzd5 | 1500 | 1501 | Fzd5 | matched |
| 1428664_at | SCN | Vip | 77371835 | 1103 1102 | Vip | matched |
| 1450428_at | SCN | Lhx1 | 633 79591731 | 79904282 634 | Lhx1 | matched |
| 1450468_at | SCN | Myoc |  | 71234527 | Myoc | unconfirmable |
| 1423410_at | MPA | Meig1 |  | 80527163 | Meig1 | unconfirmable |
| 1418756_at | MPA | Trh | 71016631 | 68667258 | Trh | mismatched |
| 1437397_at | MPA | Prlr | 1268 | 1269 | Prlr | mismatched |
| 1416236_a_at | SO | Eva1 |  | 68301282 | Mpzl2 | unconfirmable |
| 1443921_at | SO | Ranbp3l |  | 69530792 | Ranbp3l | unconfirmable |
| 1448529_at | SO | Thbd | 75079800 | 75694254 | Thbd | unconfirmable |
| 1430785_at | SPa ventral | Sdro |  | 69835625 | Rdh20 | unconfirmable |
| 1446438_at | SPa ventral | Ubr1 | 74957908 | 70194540 | Ubr1 | unconfirmable |
| 1439190_at | SPa dorsal | Fhad1 |  | 68546125 | Fhad1 | unconfirmable |
| 1418315_at | SPa dorsal | Nr5a1 | 734 | 735 | Nr5a1 | matched |
| 1417760_at | SPa dorsal | Nr0b1 |  | 77464954 | Nr0b1 | matched |
| 1420471_at | DM | Hcrt | 2283 | 2284 79913316 | Hcrt | matched |
| 1421686_at | DM | Rfrp | 74882808 | 69818107 | Npvf | matched |
| 1427550_at | DM | Peg10 | 74357549 | 73497633 | Peg10 | mismatched |
| 1457048_at | VMH | Gpr103 | 71717619 | 70724756 | Gpr103 | unconfirmable |
| 1450018_s_at | VMH | Slc25a30 |  | 69095952 | Slc25a30 | unconfirmable |
| 1430329_at | VMH | Cgn |  | 71579885 | Cgn | unconfirmable |
| 1433800_a_at | Arc | Pomc1 | 2493 80517122 | 2494 | Pomc | matched |
| 1448326_a_at | Arc | Crabp1 | 1179 | 1180 | Crabp1 | matched |
| 1419634_a_at | Arc | Ghrh | 496 | 497 | Ghrh | mismatched |
| 1450610_at | PAG | Ucn | 1435 | 1436 1437 | Ucn | matched |
| 1435750_at | PAG | Gchfr | 74511806 | 69672820 | Gchfr | mismatched |
| 1435670_at 1423340_at | SC | Tcfap2b |  | 70598361 | Tcfap2b | unconfirmable |
| 1444596_at | SC | Pax7 | 766 | 767 | Pax7 | mismatched |
| 1425443_at | IC | Tcfap2d |  | 69290633 | Tcfap2d | unconfirmable |
| 1425840_a_at | IC | Sema3f | 72007563 | 69095978 | Sema3f | mismatched |
| 1418496_at | VTA | Foxa1 | 2228 77869794 | 2229 77800889 | Foxa1 /// LOC100047556 | matched |
| 1440803_x_at 1437029_at | VTA | Tacr3 | 1297 | 80342167 1298 | Tacr3 | matched |
| 1421677_at | SN | Fgf20 |  | 434 435 | Fgf20 | unconfirmable |
| 1460129_at | Tg | Slc6a2 | 73615562 | 70785098 | Slc6a2 | matched |
| 1419628_at | Tg | Chx10 | 1687 | 1688 | Vsx2 | unconfirmable |
| 1425409_at | Tg | Chrna2 | 75551460 | 75551480 | Chrna2 | mismatched |
| 1450734_at | Pn | Lztr2 |  | 69015003 | Sec16b | matched |
| 1444451_at | Pn | Pappa2 | 276066 | 199143 | Pappa2 | unconfirmable |
| 1456229_at | MVe | Hoxb3 |  | 69289153 | Hoxb3 | unconfirmable |
| 1455907_x_at | MVe | Phox2b | 2480 | 2481 79904242 | Phox2b | matched |
| 1449865_at | MVe | Sema3a | 79591337 937 | 79761021 938 | LOC100044161 /// Sema3a | matched |
| 1458084_at | Cb vermis | Zdhhc17 |  | 70303468 | Zdhhc17 | unconfirmable |
| 1424944_at | Cb lobe | Pcp2 | 655115 79490073 77413702 | 74357592 | Pcp2 | matched |
| 1455851_at | Cb lobe | Bmp5 | 73817428 | 69529364 | Bmp5 | unconfirmable |
| 1415832_at | Cb nucleus | Agtr2 |  | 71608203 | Agtr2 | mismatched |
| 1430335_a_at | Cb nucleus | Pax3 | 2464 | 2465 | Pax3 | mismatched |
| 1436704_x_at | Cb nucleus | Mthfd1 |  | 71763804 | Mthfd1 | unconfirmable |

The list provides the Affymetrix probe set ID (“BrainStars probe set ID”), highly expressed region, Allen Brain Atlas (ABA) gene symbol, ABA image series ID (coronal), ABA image series ID (sagittal), gene symbol, and the result of the comparison.
