# Supplementary material for: Quantitative Expression Profile of Distinct Functional Regions in the Adult Mouse Brain
Source: PLoS One. 2011 Aug 12;6(8):e23228. doi: 10.1371/journal.pone.0023228 (PMC3155528; doi:10.1371/journal.pone.0023228)
Supplement: Text S1 — Supporting materials and methods, and results. (DOC) [file pone.0023228.s009.doc]

# Supporting information for Quantitative Expression Profile of Distinct Functional Regions in the Adult Mouse Brain

Takeya Kasukawa, Koh-hei Masumoto, Itoshi Nikaido, Mamoru Nagano, Kenichro D. Uno, Kaori Tsujino, Carina Hanashima, Yasufumi Shigeyoshi, and Hiroki R. Ueda

## Supporting Results and Discussion

**Similar expression between the globus pallidus (GP) and substantia nigra (SN)**

The globus pallidus (GP) and substantia nigra (SN), which are classified into different classical developmental/evolutional/anatomical divisions, the telencephalon and mesencephalon, respectively, but are functionally and anatomically linked, exhibited a tight and robust clustering of their genome-wide expressions in correlation and Euclidean distances (**Figure 1C and Figure S1C**–**E**). Developmentally, GP and SN are derived from different origins in the mammalian development. GP is located in the rostral forebrain, which originates in the ventral part of the telencephalon that is derived from the alar plate (dorsal part of the neural tube). SN is located in the midbrain, which originates in mesencephalon that is derived from the basal plate (ventral part of the neural tube) [1]. From an evolutionary perspective, GP is a relatively new invention created in cartilaginous fish and did not exist in the CNS prototype of cyclostomes [2]. Hence, based on classical developmental and evolutionary perspectives, it was surprising to see the similar expression between GP and SN.

Anatomically, SN consists of two regions, Pars compacta and Pars Reticulata. SN Pars compacta consists of well-known dopamine neurons (A9 neuron) whereas SN Pars Reticulata is comprised of different types of neurons that probably originate from different regions of the mesencephalon [3]. However, these two SN regions are strongly connected to the basal ganglia, functionally forming one system. This functional system includes SN Pars Reticulata, Subthalamic nucleus and GP, which are all mutually dependent on each other by carrying out integrated information processing. These anatomical and functional linkages suggest that the similarities observed in expression could be predicted by anatomical and functional linkage. However, we note that anatomical and functional linkages alone do not predict the similarity of expression between two CNS regions because the striatum (ST), which is functionally linked to the GP and also more closely located to the GP than the physical distance between the GP and SN [3], exhibits a more dissimilar expression to GP. Therefore, the similarity in expression between the GP and the SN was an unexpected finding that could not be predicted from only classical developmental/evolutional/anatomical knowledge.

What is the origin of the similar expression observed between these two CNS regions? At the molecular level, we note that the development of the mesencephalon region deriving the SN is promoted by *Sonic hedgehog* (*Shh*). Similarly, the development of the ventral part of the telencephalon, leading to the rostral and eventually the GP, is also promoted by *Shh* [4]. Considering that GP is a new addition since the appearance of cartilaginous fish, one attractive evolutionary-developmental biological hypothesis would be that the prototypic genetic program characterizing the origin of SN (especially Pars Reticulata) had been copied and pasted to invent the GP upon the linkage to the positional information in the basal ganglia, and hence, the dependence on *Shh* may also had been copied. Supporting this idea, *Nkx2.1* specifies the development of the neuronal precursor cells of the GP but not those of the ST whereas *Nkx2.2*, a close relative of *Nkx2.1*, is widely expressed along the anterior-posterior axis of the basal plate (ventral part of neural tube), which derives the mesencephalon and eventually the SN [5,6].

**Functions of stable and variabile one-state genes**

We observed that the stable and variable expression patterns of the one-state genes seemed correlate with their subcellular localizations (**Figure S3M–P**).  For example, genes whose product function in the nucleus tended to be stable (**Figure S3M**), whereas those whose product are located in the plasma membrane or extracellular region tended to be variable (**Figure S3O–P**). On the other hand, genes encoding cytoplasmic products tended to show intermediate (i.e. without significance) variability (**Figure S3N**). These findings concur with a recent report correlating protein function with intracellular location [7].  We also observed that the molecular functions of gene products seem to correlate with the stability and variability of their one-state gene expression (**Figure S3Q–R**), as exemplified by genes for nucleic acid-binding proteins being enriched among the stable one-state genes (**Figure S3Q**), whereas those for protein-binding proteins were enriched among the variable one-state genes (**Figure S3R**). In addition, some biological processes showed correlations with the stability and variability of one-state gene expression (**Figure S3S–T**); genes for metabolic process proteins were enriched among the stable one-state genes, whereas those encoding proteins involved in developmental processes were enriched among the variable one-state genes.

**Comparison of the BrainStars dataset with Allen Brain Atlas**

We also compared the BrainStars dataset with the ABA dataset at the reproducible and quantitative points. We collected a larger number of mice for the sampling of each CNS (central nervous system) region to reduce the variation between experimental replicates. In detail, we sampled 5–25 mice for each CNS region at each time point (every 4 hours over one day), resulting in samples from 30–150 mice being collected for each replicate of a single CNS region. This entire procedure in the BrainStars project was performed twice to obtain experimental replicates for every CNS region. This sampling strategy helped to reduce the expression variability between experimental replicates of the same brain region, as shown in **Figure S5A**. This scatter plot shows the high reproducibility of the BrainStars project, in which the correlation coefficient was 0.994 for the experimental replicates for all the CNS regions. In contrast, the entire procedure in the ABA project was only performed once. *In situ* hybridization (ISH) experiments in the ABA project were repeated at least twice for only approximately 7.7% of all the probes. **Figure S5B** shows the reproducibility for such probes in the ABA (correlation coefficient = 0.694), which was much lower than the reproducibility in the BrainStars. We also note that the dynamic range, which we defined as the range of the more than 50% of replicated data points that showed a less-than 2-fold change, was from 2^4^ to 2^14^ (approximately 10^3.0^-fold) for the BrainStars project and from 2^2^ to 2^5^ (approximately 10^0.9^-fold) for the ABA project, respectively **Figure S5C–D**. To confirm the reproducibility in the overall comparison of 51 CNS regions, we performed detailed comparisons of each CNS region, and again found higher reproducibility in the BrainStars than in the ABA project. In detail, the maximum, median, and minimum correlations in the BrainStars project between the experimental replicates for each CNS region were 0.997, 0.995, and 0.975, respectively (**Figure S5E**), whereas the corresponding figures for the ABA project were 0.774, 0.695, and 0.434, respectively (**Figure S5F**). These results suggest that the expression data in the BrainStars project have higher reproducibility and a greater dynamic range than those of the ABA project. To confirm the high reproducibility in the BrainStars project, we also performed hierarchical clustering of the 102 samples with the non-control probe set, and found that almost all of the experimentally replicated samples were closely clustered (**Figure S1A**). Although we acknowledge that there were misclassifications of a few CNS regions in this hierarchical clustering of the BrainStars project, these seemed to occur because of the similar overall expression patterns of the corresponding CNS regions, and therefore were likely inherent limitations of the clustering method. For example, we found that the Cx cingulate (cerebral cortex cingulate) and Cx motor (cerebral cortex motor) were mis-clustered together. However, even for these mis-clustered CNS regions, when we looked at the appropriate set of regional genes, their expression pattern in our data resource could correctly distinguish the corresponding CNS regions, as shown for *Myl4* (**Figure S5G**), which is also regionally expressed in the ABA (http://mouse.brain-map.org/brain/Myl4.html). These results also support the high reproducibility of the BrainStars project. We also note that the dynamic range is much higher in the BrainStars than the ABA dataset.

The number of independent probes for each gene and the consistency of expression detected by these redundant probes in the BrainStars project were also much higher than in the ABA. In fact, we carefully compared the actual number of redundant probe sets as well as the ratio of highly correlated redundant probes in both datasets, and found that both figures were higher in the BrainStars project than in the ABA project. For example, there are 7,233 probe sets in the BrainStars dataset, whereas there are only 4,494 probe sets in the ABA dataset. We also found that 2,621 (36.2%) of the redundant probe sets exhibited a high correlation (>0.8) in the BrainStars dataset, while only 760 (16.9%) did so in the ABA dataset. In detail, the BrainStars dataset contains 45,037 non-control oligo-probes corresponding to 34,323 target transcripts (**Figure S5H**), of which 7,233 target transcripts have redundant probe sets. In contrast, the ABA dataset contains 23,722 ribo-probes corresponding to 20,773 target transcripts (**Figure S5H**), of which 4,494 target transcripts have redundant probe sets. Therefore, the number of target transcripts with redundant probe sets is greater in the BrainStars than in the ABA dataset. To evaluate the overall consistency of expression detected by redundant probe pairs, we calculated the maximum Pearson product-moment correlation coefficient among each redundant probe set (**Figure S5H**). The ratio of highly correlated redundant probes (correlation > 0.8) in the BrainStars dataset was 2,621 (36.2%) of 7,233 redundant probe sets, which is higher than the 760 (16.9%) of 4,494 redundant probe sets in the ABA dataset. To evaluate the consistency of the expression detected by the redundant probe sets in detail, we calculated the maximum correlation among redundant probe sets, and plotted their distribution for both datasets (**Figure S5I–J**). The BrainStars dataset had peaks of maximum correlation around 0.9-1.0 (**Figure S5I**), whereas the ABA dataset had a peak of maximum correlation around 0-0.1 and 0.7-0.8 (**Figure S5J**), implying that a larger number of redundant probes did not correlate well in the ABA dataset. These results indicated that the consistency of expression detected by redundant probe sets was higher in the BrainStars dataset. In conclusion, the number of redundant probes for each gene, as well as the consistency of expression detected by redundant probes in the BrainStars dataset were higher than in the ABA dataset.

After performing careful comparisons, we strongly believe that the quantitativeness and reproducibility of BrainStars are higher than compared with those of the ABA. We respect the Allen Brain Atlas database (a high-throughput whole-brain ISH database), especially for its single-cell-level spatial resolution. However, the ABA also has issues in its reproducibility and consistency of redundant probe sets, probably due to its lower dynamic range, as described above. We thus believe that the complementary and cooperative usage of these genome-wide expression datasets, would be a useful platform for further investigation of the structure and function of the adult mouse brain.

## Supporting Materials and Methods

**Identification of multi-state genes**

Genes with multi-state expression patterns were identified with a variational Bayesian inference to fit a Gaussian mixture model [8]. We used log_2_-transformed expression values for the 45,037 non-control probe sets in the 48 CNS regions that did not include the retina, pituitary, or pineal.

Before identifying genes with multi-state expression patterns, we chose a training set for this analysis in which the expression levels of genes were clearly higher or lower in a single CNS region than in the others. These genes were identified based on the template-matching method [9].  First, for each region, we made a template pattern with 1 as the target region and 0 for the others, and calculated Pearson's correlation coefficients between the template and log_2_-transformed expression values of each probe set.  We then performed statistical tests for all the correlation values using Fisher's Z-transform to calculate the *P*-values, and corrected the multiple comparisons by assigning a false discovery rate (*FDR*) to them.  After the test, we retrieved the probe sets for which the *FDR* of the Pearson's correlation between the template of the highest (or the lowest) region and the log_2_-transformed expression values was less than or equal to 0.0001.

To identify multi-state genes in the adult mouse brain, we modeled the distribution of the log_2_-transformed expression values of a single probe set $X=\{x_{n}|n=1..N\}$ in *N* brain regions (*N =* 48, in this study) using the following Gaussian mixture model [8]:

$p(x)=\sum_{k=1}^{K} \pi_{k}N(x|\mu_{k},\lambda_{k}^{-1})$
where *K* is the number of components (or “states”) in the Gaussian mixture, *N*(•) is the Gaussian distribution, and the parameters $\mu_{k},\lambda_{k},\pi_{k}$ are the mean, precision (=1/variance), and mixing coefficient (weight) of a mixture component *k*. To estimate these parameter values, we performed variational Bayesian inference. In detail, conjugate prior distributions of the parameters $\mu_{k},\lambda_{k},\pi_{k}$ were defined as follows:
$p(\mathbf{\pi})=\text{Dirichlet}(\mathbf{\pi}|\alpha_{0})$, $p(\mu_{k},\lambda_{k})=N(\mu_{k}|m_{0},(\beta_{0}\lambda_{k})^{-1})\text{Gamma}(\lambda_{k}|a_{0},b_{0})$
where $\pi=\{\pi_{k}\}$, and Dirichlet(•) and Gamma(•) are the Dirichlet and gamma distributions, respectively. $\alpha_{0}=\{\alpha_{0}\},m_{0},\beta_{0},a_{0},b_{0}$ are prior hyper-parameters. The prior hyper-parameter $\beta_{0}$ is defined as 0.001, which was intended to be a non-informative prior distribution, and $m_{0}$is the mean of *X*. To determine the prior hyper-parameters $a_{0}$ and $b_{0}$, the median of error variances ($v_{0}$) of the duplicated expression values (n = 2) of 48 brain regions was calculated for all probe sets. Since the distribution of $1/v_{0}$ can be fitted to a gamma distribution (**Figure S2A**), $a_{0}$ was determined from the mean and the variance of the distribution of $1/v_{0}$ as ~2.233 (= mean^2^/variance). $b_{0}$ was defined as $v_{0}$×$a_{0}$ for each probe set. Finally, to determine the prior hyper-parameter $\alpha_{0}$, the subsequent variational Bayesian procedures were performed 2,000 times by changing $\alpha_{0}$ to 1, 0.5, 1/3, 0.25, 0.2, 1/6, and 0.1, and the sums of the false-positive and false-negative rates of the training set were calculated (**Figure S2B**). By this procedure, $\alpha_{0}=1/3$, which has the minimum sum of false-positive and -negative rates, was chosen as a prior hyper-parameter.

To infer the posterior distributions,, and
where are posterior hyper-parameters, the following procedure was performed: 1) the initial values for hyper-parameters were set; 2) the following values were calculated:


,
where is a digamma function. 3) This calculation was repeated until the lower bound (*L*) did not improve (difference between the previous and current *L* < 1.0×10^-6^):

$\begin{matrix} L=-\frac{1}{2}N\ln(2\pi)-\sum_{k=1}^{K} \sum_{n=1}^{N} r_{nk}\ln r_{nk}+\ln\frac{\Gamma\left(\sum_{k=1}^{K} \alpha_{0}\right)}{\prod_{k=1}^{K} \Gamma(\alpha_{0})}-\ln\frac{\Gamma\left(\sum_{k=1}^{K} \alpha_{k}\right)}{\prod_{k=1}^{K} \Gamma(\alpha_{k})}+\frac{1}{2}\sum_{k=1}^{K} \ln\left(\frac{\beta_{0}}{\beta_{k}}\right) \\ \text{ }+\sum_{k=1}^{K} \left\{\ln\left(\frac{\Gamma(a_{k})}{\Gamma(a_{0})}\right)+a_{0}\ln b_{0}-a_{k}\ln b_{k}\right\} \end{matrix}$.
The procedure was iterated 50,000 times. We determined this number (50,000) by tracking the number of effective components, which means that at least one data point (region) was clustered into the components, for every 200 repeats in this procedure, and by judging that the inference of our data set was converged. After the iteration, the result with the lowest *L* was chosen as the best-fit parameters of the Gaussian mixture model by$\mu_{k}=m_{k}$, $\lambda_{k}=a_{0}/b_{0}$, $\pi_{k}=\frac{\alpha_{k}}{\sum_{j=1}^{K} \alpha_{j}}$. Each brain region *n* was then assigned to a “state” *k* that corresponded to the mixture component *k* with the maximum $r_{nk}$ value in $\{r_{nk}|k=1..K\}$. Sometimes two or more mixture components overlapped, causing a situation where one state was nested into another state (**Figure S2C**). For this situation, if regions *n*1 and *n*2 in state 1, and a region *n*3 in state 2 satisfied *x_n_*_1_ < *x_n_*_3_ < *x_n_*_2_, states 1 and 2 were merged into a single state.

After 50,000 repeated fitting procedures and choosing the best-fit result for each probe set, the CNS regions were grouped according to predicted states. For example, CNS regions were classified into three groups for three-state genes because there were three states (“high,” “low,” and “middle”).

**Functional analysis on one-state genes**

Gene Ontology assignments to the probe sets were retrieved from the Bioconductor 2.3 annotation packages [10]. To identify the functions of the protein products of genes that were enriched among the variable or stable genes, we assigned a *P*-value to every GO term using the Wilcoxon rank-sum test between the variability scores of genes that were assigned the GO term and those that were not. After the *P*-value assignment, we calculated the FDR.

## Supporting References

1. Paxinos G (2004) The Rat Nervous System, Third Edition. San Diego, CA: Academic Press.

2. Reiner A, Medina L, Veenman CL (1998) Structural and functional evolution of the basal ganglia in vertebrates. Brain Res Brain Res Rev 28: 235-285.

3. Kandel ER, Schwartz JH, Jessell TM (2000) Principles of Neural Science. New York: McGraw-Hill Medical.

4. Ericson J, Muhr J, Jessell TM, Edlund T (1995) Sonic hedgehog: a common signal for ventral patterning along the rostrocaudal axis of the neural tube. Int J Dev Biol 39: 809-816.

5. Sussel L, Marin O, Kimura S, Rubenstein JL (1999) Loss of Nkx2.1 homeobox gene function results in a ventral to dorsal molecular respecification within the basal telencephalon: evidence for a transformation of the pallidum into the striatum. Development 126: 3359-3370.

6. Shimamura K, Hartigan DJ, Martinez S, Puelles L, Rubenstein JL (1995) Longitudinal organization of the anterior neural plate and neural tube. Development 121: 3923-3933.

7. Ertel A, Tozeren A (2008) Switch-like genes populate cell communication pathways and are enriched for extracellular proteins. BMC Genomics 9: 3.

8. Bishop CM (2006) Pattern Recognition and Machine Learning Jordan M, Kleinberg J, Schölkopf B, editors. New York: Springer.

9. Pavlidis P, Noble WS (2001) Analysis of strain and regional variation in gene expression in mouse brain. Genome Biol 2: RESEARCH0042.

10. Gentleman RC, Carey VJ, Bates DM, Bolstad B, Dettling M, et al. (2004) Bioconductor: open software development for computational biology and bioinformatics. Genome Biol 5: R80.

**Table S1. Sample information for all 51 central nervous system (CNS) regions.**

**Table S2. Pearson’s correlation coefficients of the BrainStars dataset with the Allen Brain Atlas (ABA) dataset.**

**Table S3. Comparison of the marker gene candidates in the BrainStars dataset with the Allen Brain Atlas (ABA) dataset.**
